# Patterns of structural variation define prostate cancer across disease states

**DOI:** 10.1172/jci.insight.161370

**Published:** 2022-09-08

**Authors:** Meng Zhou, Minjeong Ko, Anna C.H. Hoge, Kelsey Luu, Yuzhen Liu, Magdalena L. Russell, William W. Hannon, Zhenwei Zhang, Jian Carrot-Zhang, Rameen Beroukhim, Eliezer M. Van Allen, Atish D. Choudhury, Peter S. Nelson, Matthew L. Freedman, Mary-Ellen Taplin, Matthew Meyerson, Srinivas R. Viswanathan, Gavin Ha

**Affiliations:** 1 Department of Medical Oncology, Dana-Farber Cancer Institute, Boston, Massachusetts, USA.; 2 Broad Institute of MIT and Harvard, Cambridge, Massachusetts, USA.; 3 Harvard Medical School, Boston, Massachusetts, USA.; 4 Public Health Sciences and Human Biology Divisions, Fred Hutchinson Cancer Center, Seattle, Washington, USA.; 5 Department of Pathology, UMass Memorial Medical Center, Worcester, Massachusetts, USA.; 6 Department of Cancer Biology, Dana-Farber Cancer Institute, Boston, Massachusetts, USA.; 7 Center for Cancer Genomics, Dana-Farber Cancer Institute, Boston, Massachusetts, USA.; 8 Department of Genome Sciences, University of Washington, Seattle, Washington, USA.; 9 Center for Functional Cancer Epigenetics, Dana-Farber Cancer Institute, Boston, Massachusetts, USA.

**Keywords:** Genetics, Oncology, Bioinformatics, Genetic variation, Prostate cancer

## Abstract

The complex genomic landscape of prostate cancer evolves across disease states under therapeutic pressure directed toward inhibiting androgen receptor (*AR*) signaling. While significantly altered genes in prostate cancer have been extensively defined, there have been fewer systematic analyses of how structural variation shapes the genomic landscape of this disease across disease states. We uniformly characterized structural alterations across 531 localized and 143 metastatic prostate cancers profiled by whole genome sequencing, 125 metastatic samples of which were also profiled via whole transcriptome sequencing. We observed distinct significantly recurrent breakpoints in localized and metastatic castration-resistant prostate cancers (mCRPC), with pervasive alterations in noncoding regions flanking the *AR*, *MYC*, *FOXA1*, and *LSAMP* genes enriched in mCRPC and *TMPRSS2-ERG* rearrangements enriched in localized prostate cancer. We defined 9 subclasses of mCRPC based on signatures of structural variation, each associated with distinct genetic features and clinical outcomes. Our results comprehensively define patterns of structural variation in prostate cancer and identify clinically actionable subgroups based on whole genome profiling.

## Introduction

Over the past decade, genomic sequencing studies have progressively sharpened our view of the genetic landscape of prostate cancer (1). Such studies have defined key driver genes in prostate cancer and have enabled the deployment of therapeutic agents in molecularly defined disease subsets, including potent androgen receptor–targeted (*AR*-targeted) therapies (2, 3), poly(ADP-ribose) polymerase (PARP) inhibitors in *BRCA1/2*-altered prostate cancers, and immune checkpoint inhibitors in cancers with microsatellite instability (4–7).

To date, most cancer genomic studies have used whole exome sequencing (WES) and have thus been focused on mutations or copy number alterations that occur within the protein-coding regions of genes, which represent only 1%–2% of the genome. More recent studies applying whole genome sequencing (WGS) to prostate and other cancers have identified previously underappreciated recurrent alterations in regulatory (noncoding) regions of the genome and have illuminated complex mechanisms of genomic alterations — driven by structural variants (SVs) — that are difficult to discern by WES; in the case of prostate cancer, most of these studies have focused on localized disease, the disease state in which tissue is most readily accessible for profiling (8–22). There remains a need for continued high-resolution genomic discovery efforts in prostate cancer.

In addition to efforts characterizing entire cancer genomes, recent studies have illustrated the importance of molecularly profiling prostate cancer across disease states. While many localized prostate cancers can be cured with surgery or radiotherapy, a substantial portion of higher-risk cancers recur and progress to metastatic disease, which is incurable. Recurrent prostate cancer may have a long natural history, during which time a patient may receive several lines of therapy — with androgen deprivation therapy (ADT) as a backbone — that may shape the cancer’s genomic landscape (23).

Indeed, while hormone-refractory castration-resistant prostate cancer (CRPC) has been less extensively profiled than localized prostate cancer, several studies have indicated that CRPCs display genomic landscapes distinct from treatment-naive disease (24, 25). A cardinal hallmark of CRPC is the reactivation of *AR* signaling in the face of maximal ADT (22, 26–28). This may occur via diverse mechanisms, including the production of constitutively active *AR* splice variants (*AR-V*s) and activating mutations or copy number amplifications of the *AR* gene (29–31) or of regulatory elements distal to the gene body (13, 15, 32). To date, the relative contribution of each of these mechanisms in driving *AR* reactivation in CRPC has not been systematically explored. Also needed is a more global map of significant hotspots of structural variation in prostate cancer genomes, drawn within a rigorous statistical framework.

In this study, we performed linked-read WGS on 36 metastatic castration-resistant prostate cancer (mCRPC) tumor–normal pairs. We combined these data with WGS (101 metastatic cases and 531 localized cases) and whole transcriptome sequencing (RNA-Seq, 99 cases) data from previously described localized and metastatic CRPC cohorts (9, 13, 15, 33). We then established a harmonized workflow for the integrative genomic analysis of 531 localized and 143 metastatic CRPC samples, interrogated both hotspots and genome-wide patterns of structural variation, and evaluated their consequences.

## Results

### WGS analysis of localized and metastatic prostate cancer cohorts.

**Figure 1:**
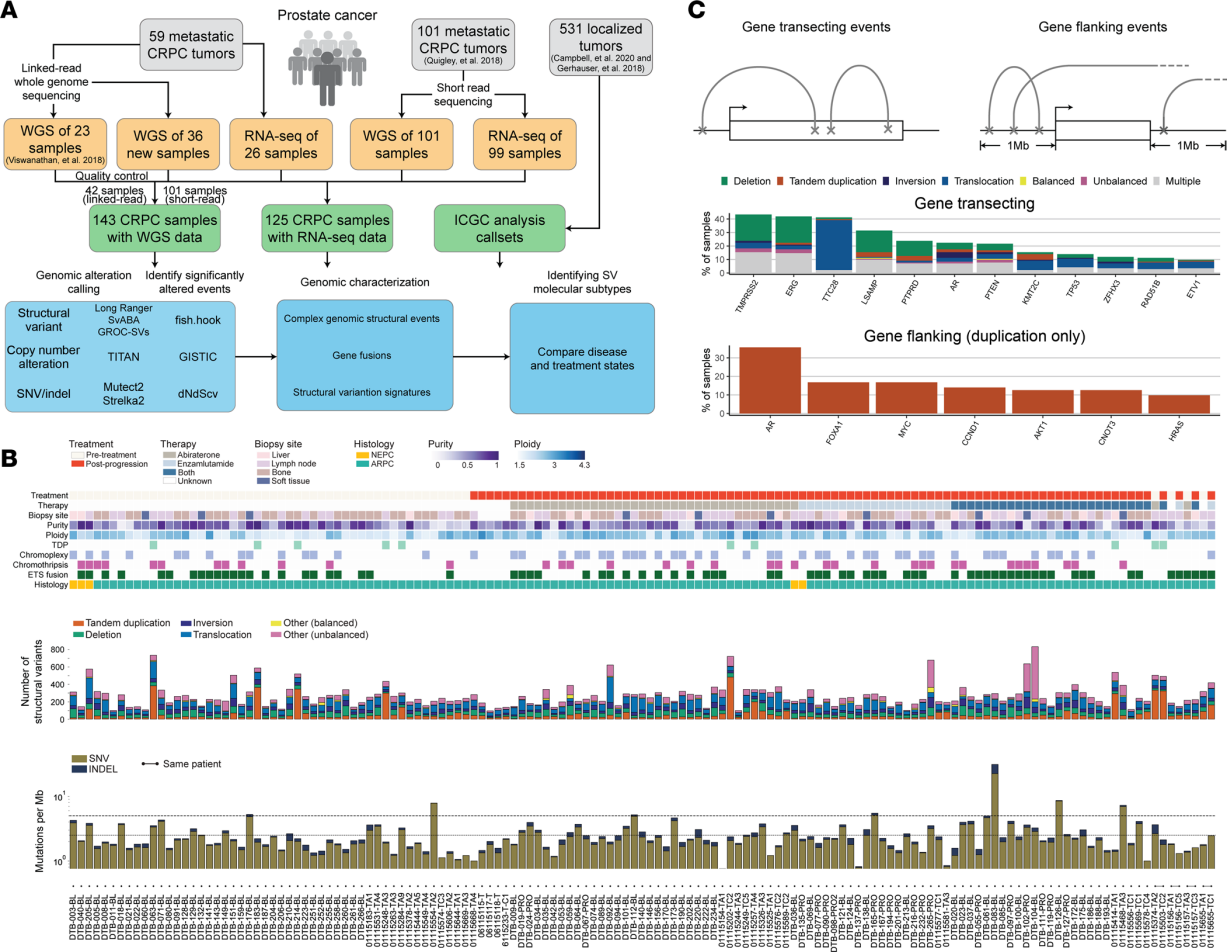
Study overview of prostate cancer across disease stages and the genomic landscape of mCRPC. (**A**) Workflow of study and data analysis. Tumor specimens (gray) from both primary prostate cancer and mCRPC were included in this study. Linked-read and short-read WGS and RNA-Seq data sets were either generated for this study or reanalyzed from prior studies (9, 33). An overview of the genomic alteration and characterization analysis is shown. (**B**) Clinical annotations and somatic alterations for 143 patient samples in the pooled mCRPC cohort. Samples are ordered by treatment type; the 4 patients with pretreatment and post-progression pairs are placed at the right. Top: Clinical and sample information and genomic pattern classifications, including neuroendocrine prostate cancer (NEPC) and androgen receptor pathway active prostate cancer (ARPC). Middle: Distribution of genomic rearrangement types in individual samples. Bottom: Mutational burden for SNVs and indels computed as number of mutations per mega–base pair (Mb). *Y* axis shown in logarithmic scale. Threshold lines indicate mutational burden at 2.5 and 5 mutations per Mb. (**C**) Genomic rearrangement alteration profiles of key mCRPC genes. Top: Events were categorized into gene transecting or gene flanking based on the overlap of breakpoints with the gene body and flanking 1 Mb of either the transcription start site or the termination site of the gene. Only 159 genes reported and known to be involved in prostate cancer were considered in this analysis (Supplemental Table 1, G and H). Middle: Frequency and distribution of rearrangement types for gene transecting events; genes with ≥10% frequency are shown. Gene transecting events were prioritized over flanking events during annotation. The category “Multiple” represents gene-sample pairs carrying more than 1 type of rearrangement event. Bottom: Frequency of gene flanking events by tandem duplication; genes with ≥10% are shown.

We performed linked-read WGS on 36 biopsy specimens from 33 mCRPC patients and matched blood normal controls. After quality control, 17 tumor samples were excluded based on insufficient tumor purity and/or contamination, reflecting the challenge of obtaining high-purity metastatic biopsies, particularly from bone lesions (34). We included only samples with tumor purity greater than 15% in downstream analyses so as to increase confidence in SV calls (Figure 1A and Supplemental Table 1; supplemental material available online with this article; https://doi.org/10.1172/jci.insight.161370DS1). We reanalyzed a linked-read WGS data set of 23 samples published previously by our groups (15), resulting in a total of 42 linked-read WGS samples from 38 patients with mean coverage of 34× (range 21× to 54×) and 33× (range 25× to 45×) for tumor and normal samples, respectively (Supplemental Table 1A). The mean molecule length was 29 kb and 34 kb in tumor and normal samples, respectively (Supplemental Table 1A).

We further combined these data with 101 mCRPC samples sequenced with standard short-read sequencing, published previously (13). This resulted in the generation of a final combined cohort of 143 tumor-normal pairs, which were uniformly analyzed for copy number and structural alterations via a harmonized pipeline (Figure 1A). Of these, 125 also had RNA-Seq data available (26 from the linked-read cohort and 99 from the standard short-read WGS cohort). Fifty-four samples (37.8% of 143 samples) were collected at castration resistance, prior to treatment with a second-generation androgen receptor signaling inhibitor (ARSi) such as abiraterone and/or enzalutamide (“pretreatment”), while the remaining 89 samples (62.2% of 143 samples) were collected at progression (“post-treatment”; Figure 1B and Supplemental Table 1B). We analyzed the somatic single-nucleotide variants (SNVs), insertion-deletions (indels), copy number alterations (CNAs), and SVs in the combined cohort and identified recurrent somatic alterations in each of these classes (Figure 1A).

A total of 2,315,452 SNVs and indels were called, with a mean tumor mutation burden of 2.82 mutations per million bases (Mb). We confirmed that known driver genes of prostate cancer were enriched for non-synonymous mutations, including *TP53*, *RB1*, *PTEN*, *FOXA1*, *CDK12*, *AR*, and *SPOP* among known Catalogue of Somatic Mutations in Cancer (COSMIC) Cancer Gene Census genes (dNdScv, *q* ≤ 0.1; Supplemental Table 1, C and D). We detected an average of 272 (range 96–833) SV events per sample. Based on breakpoint orientations, SV events were classified into deletions, inversions, tandem duplications, interchromosomal translocations, and intrachromosomal translocations, while intrachromosomal translocations were further divided into balanced and unbalanced events based on copy number information. Chromoplexy was detected in 53 samples (37.1% of 143 samples), while chromothripsis was detected in 37 samples (25.9%); these events were not mutually exclusive (Fisher’s exact test, log-odds ratio = 1.417, *P* = 0.612). Ten cases (7.0%) harbored a genome-wide tandem duplicator phenotype (TDP), all of which had *CDK12* inactivating alterations, as recently reported (15, 35). We found that TDP was mutually exclusive with E26 transformation–specific (ETS) rearrangements (Fisher’s exact test, log-odds ratio = 0.133, *P* = 0.043) and chromothripsis (log-odds ratio = 0.301, *P* = 0.007), as previously reported (10, 13, 15, 35).

Analysis of CNA events across the genome revealed amplification and deletion peaks in the regions of known prostate cancer genes (10, 13, 15, 24). Many oncogenic drivers of mCRPC, such as *AR* and *MYC*, were within peaks of amplification across the cohort, while tumor suppressors such as *PTEN*, *TP53*, and *KMT2C* were found within deletion peaks (Supplemental Figure 1C and Supplemental Table 1, E and F), consistent with prior reports (10, 13, 15, 36).

### Recurrent somatic structural variants in prostate cancer–associated genes.

Structural variants may either activate or inactivate gene function, depending on the location of the breakpoints and the specific class of SV. We analyzed the potential impact of SVs called across our combined cohort, distinguishing between those with predicted inactivating (“gene transecting events”) and activating (“gene flanking events”) effects (Figure 1C, Supplemental Figure 1C, and Supplemental Table 1, G and H). Frequent gene transecting alterations were observed at the *TTC28* (37.1% of 143 samples), *LSAMP* (31.5%), and *PTPRD* (23.8%) loci, which have not been extensively studied in prostate cancer, though they have been reported in callsets for certain cohorts (28). Rearrangements involving *TTC28* were predominantly interchromosomal translocations between the gene body and various nonrecurrent partner loci (Supplemental Figure 2A). This may represent retrotransposon activity, given that the *TTC28* locus harbors an active L1 retrotransposon element (37–39). Transecting SVs within the *LSAMP* and *PTPRD* genes were predominantly deletions. Both of these genes are sites of deletion/rearrangement in cancer and have been reported to function as tumor suppressors, though they have not been extensively studied in the context of prostate cancer (40–43) (Figure 1C). Notably, although gene transecting events would be predicted to disrupt individual genes, the most frequent transecting events identified via this analysis were deletion events that span the adjacent *TMPRSS2* and *ERG* genes (observed in 37.8%), which actually produces an activating*TMPRSS2-ERG* fusion.

Duplication events that flank an intact gene could activate oncogenes, either by resulting in copy number gain of the gene or by duplicating noncoding regulatory regions (13, 15). In our combined cohort, we observed recurrent tandem duplication events with breakpoints located in the flanking gene regions of several known prostate cancer oncogenes, including *AR* (35.7%), *FOXA1* (16.8%), *MYC* (16.8%), and *CCND1* (14.0%), consistent with frequencies that have been previously reported by us and others (10, 13, 15) (Figure 1C).

Certain prostate cancer driver genes were altered by multiple classes of structural alterations in both the gene body and flanking regions (e.g., *AR*, *PTEN*), while others were predominantly altered by a single alteration class (e.g., SNVs for *TP53*, intragenic translocations for *TTC28*, or flanking tandem duplications for *MYC*) (Figure 1C and Supplemental Figure 1C). Collectively, these results catalog how diverse classes of rearrangements, both within genes and in intergenic regions, alter prostate cancer genes across disease states.

### Significantly recurrent breakpoint regions in the mCRPC genome are enriched within enhancer regions and AR binding sites.

Next, we sought to identify significantly recurrent breakpoint (SRB) regions across our combined mCRPC cohort of 143 cases in a genome-wide, unbiased manner. We applied a gamma-Poisson regression approach to model the occurrences of SV breakpoints within 100 kb windows across the cohort as previously described (44). Importantly, this model nominates SRB regions likely to function as cancer drivers by accounting for 6 different categories of covariates, including sequence features (e.g., GC content), transposable elements, fragile sites, heterochromatin regions, DNase I hypersensitivity sites, and replication timing, which may increase specificity over prior studies that have accounted only for SV frequency or for breakpoint density within a genomic window (10, 13, 15, 45).

**Figure 2:**
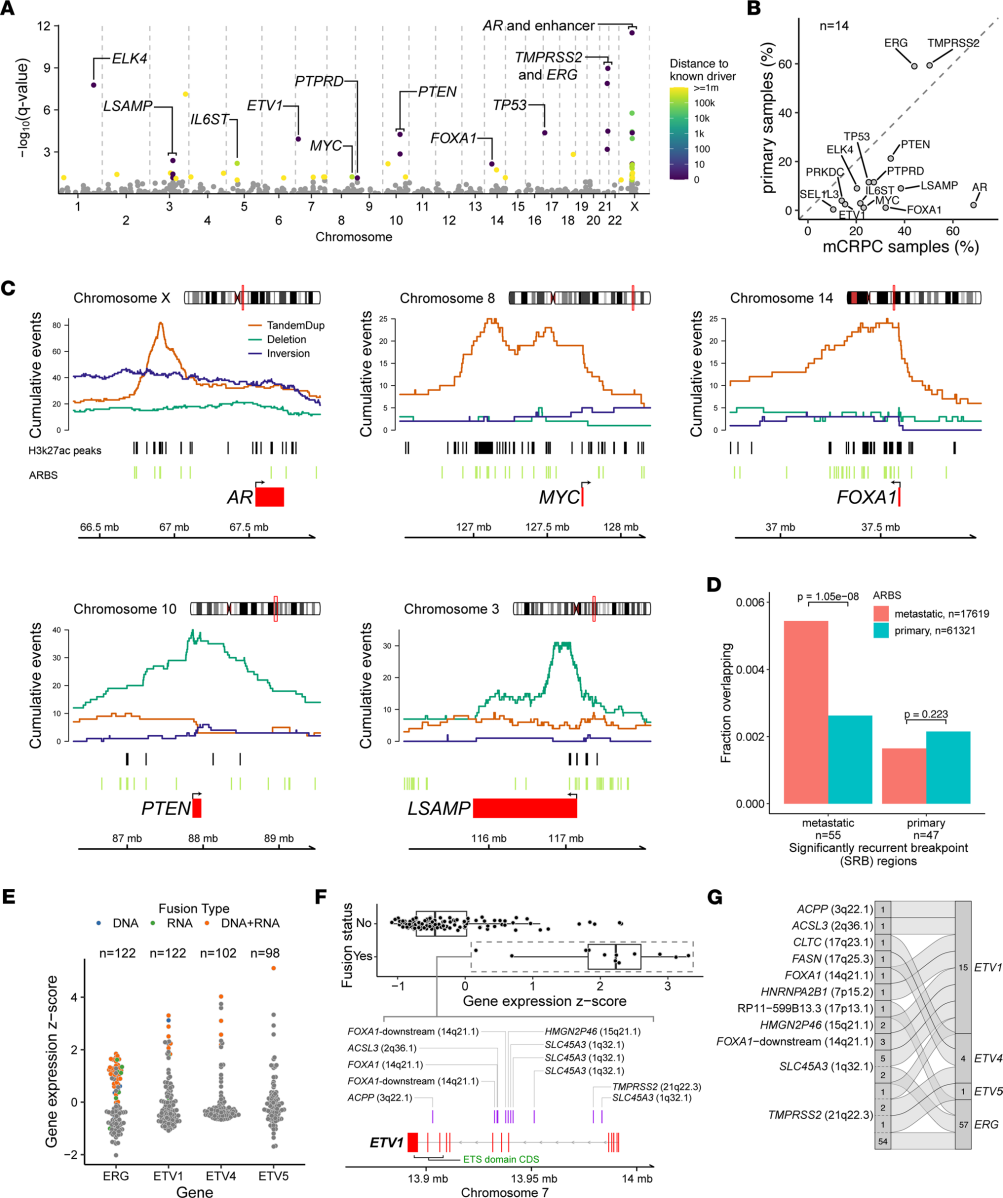
Genome-wide analysis of genomic rearrangements in mCRPC and comparisons with localized prostate cancer. (**A**) Analysis of SRBs identified regions of rearrangement hotspots, genome-wide, using a gamma-Poisson regression model. Each dot corresponds to a 100 kb bin (*n =* 26,663 total bins). Statistically significant SRB bins with FDR (Benjamini-Hochberg) *q* value ≤ 0.1 (*n =* 55) are colored based on the distance to the nearest known prostate cancer driver gene, within 1 Mb. (**B**) Comparison of SV alteration frequency in mCRPC (*n =* 143) versus primary localized prostate cancer (*n =* 278). The union set of genes (*n =* 14) within 1 Mb of SRB hotspot regions in mCRPC and localized prostate cancer cohorts was included in the comparison. (**C**) Patterns of rearrangements at the loci of driver genes identified at SRB regions in mCRPC cohort of 143 tumors. Cumulative counts of intrachromosomal SV events (tandem duplications [TandemDup], deletions, and inversions) were computed based on the breakpoints and span of the events. Interchromosomal translocations are not shown. Genome coordinates based on hg38 build. (**D**) Overlap of ARBS within SRB hotspots of mCRPC (55 regions) and primary localized prostate (47 regions) cohorts. χ^2^ test of independence *P* values is shown. (**E**) Fusion status and expression of selected genes in the ETS transcription factor gene family in the mCRPC cohort with WGS and RNA-Seq data. Fusion type was defined as the data evidence that supported the event: DNA only, corresponds to WGS; RNA only, corresponds to RNA-Seq; DNA+RNA, corresponds to support from both WGS and RNA-Seq. Each dot represents a tumor sample and is colored based on fusion type of each sample; gray indicates no evidence of fusion event. (**F**) Fusion profile of *ETV1*. DNA rearrangement breakpoints supporting the fusion (purple bars) are indicated with the corresponding fusion partners. (**G**) Summary of fusion partners for selected genes in ETS transcription factor gene family in mCRPC cohort. Fusion events and partners are indicated by flow connections. Total counts of individual fusion events and partners across the cohort are shown.

We identified a total of 55 SRB regions genome-wide across our combined mCRPC cohort (Benjamini-Hochberg corrected, *q* ≤ 0.1; Figure 2A and Supplemental Table 2A). Thirty-six (65.5%) SRB regions were located within 1 Mb of 14 known prostate cancer driver genes, including *AR* and its enhancer, *TMPRSS2*/*ERG*, *TP53*, *PTEN*, *FOXA1*, and *MYC*. For these 14 driver genes, we did not observe significant differences in SV alteration frequencies when comparing between pretreatment (*n =* 54) and post-progression (*n =* 89) samples, except in the case of *ERG*, for which the SV frequency was enriched in pretreatment samples (Fisher’s exact test, *P =* 0.0395; all other genes had *P >* 0.05; Supplemental Figure 3B). We also did not identify any major differences in the alteration frequencies of prostate cancer genes in 4 patients who had paired samples collected both before treatment with and after progression on an ARSi (Supplemental Figure 3A).

We then sought to compare how SVs drive prostate cancer across disease states. For the localized disease state, we used genome alteration calls from standard WGS of 278 primary localized prostate cancer tumors from the Pan-Cancer Analysis of Whole Genomes (PCAWG) study (9, 33). Using gamma-Poisson regression, we first identified 47 SRB regions in localized prostate cancer tumors (Supplemental Figure 2B and Supplemental Table 2B). Covariates, including LINE retrotransposons, DNase I hypersensitivity sites, and fragile sites, were statistically significant in either mCRPC or localized cohorts or both (Supplemental Table 2C). Six prostate cancer genes (*TMPRSS2*, *ERG*, *TP53*, *PTEN*, *IL6ST*, *ELK4*) within mCRPC SRB regions were also found within or in proximity (less than 1 Mb) to an SRB region in localized disease. By contrast, 4 SRBs (3 near *SEL1L3* and 1 near *PRKDC*) were unique to localized disease, while 27 SRBs were unique to mCRPC with 6 genes nearby (*LSAMP*, *ETV1*, *MYC*, *PTPRD*, *FOXA1*, *AR*). When SV alteration frequencies were compared for the 14 genes located within SRB regions in either mCRPC or localized tumors, 12 genes were significantly more altered in mCRPC samples, while *TMPRSS2* and *ERG* were significantly more altered in localized disease (Fisher’s exact test, *P <* 0.05 for all genes; Figure 2B). We repeated this comparison using an independent cohort of localized prostate cancers profiled by standard WGS (*n =* 253) and found similar genes enriched for SVs in either the localized or metastatic disease states (18) (Supplemental Figure 2, C–E). The alteration frequencies were also consistent between sequencing platforms in the mCRPC cohorts, whereby 11 and 13 of the 14 significantly enriched genes were retained when linked-read and short-read data were considered independently (Supplemental Figure 2, F and G). Thus, aggregating cancers sequenced as part of multiple distinct data sets, localized prostate cancer and mCRPC have significantly different landscapes of recurrent SVs.

To explore the potential functional consequences of SVs in intergenic SRB regions, we overlapped SV breakpoints with locations of H3K27ac marks specific to mCRPC (46). We observed that intergenic SVs within SRB regions in the mCRPC cohort included gene flanking events that were enriched at putative enhancer regions for *AR*, *MYC*, and *FOXA1*, which all had frequent focal duplication events at sites marked by mCRPC-specific H3K27ac deposition (Figure 2C and Supplemental Figure 2H). Interestingly, an intragenic deletion SRB region was observed near the transcription start site of *LSAMP*, also overlapping H3K27ac marks. *PTEN* had a high level of both gene transecting and flanking deletions, leading to SV breakpoints that were spread more broadly around the gene.

We also observed an enrichment of metastatic-specific *AR* binding sites (ARBS) compared with localized primary ARBS within the 55 mCRPC SRB regions (1-sided proportion test, *P =* 1.05 × 10^–8^; Figure 2D). This enrichment was not observed for localized primary SRB regions (*P =* 0.22). These results highlight that SVs within mCRPC SRB regions may be capturing the genome-wide footprint of activated *AR* signaling that occurs with castration resistance.

### Refined landscape of ETS gene fusions from integrated analysis of the genome and transcriptome.

We applied gene fusion analysis by integrating both genome rearrangements and fusion RNA transcript information from 127 samples with available RNA-Seq data (Figure 1A and Supplemental Table 2D). For gene fusions involving E26 transformation–specific (ETS) transcription factor gene family members (*ERG*, *ETV1*, *ETV4*, and *ETV5*), we detected 50 events supported by both DNA and RNA evidence, 15 supported by only DNA evidence, and 10 supported by only RNA evidence (Figure 2E and Supplemental Figure 2I). Overall, 74 samples (51.7% of 143 samples) harbored exactly 1 fusion event of the ETS gene family, while 1 sample had both *ERG* and *ETV1* fusion detected. In general, the frequency of ETS gene fusion was consistent with previous reports (47, 48) (Figure 1B and Supplemental Table 2D).

Among the ETS fusions, *ERG* was most commonly involved with *TMPRSS2* as the fusion partner (54 of 57 cases; Figure 2G). Other common ETS fusion partners were *SLC45A3* (7 cases) and lncRNA RP11-356O9.1 downstream of *FOXA1* (3 cases). *ETV1* had 8 distinct fusion partners, which is consistent with previous reports that *ETV1* is a promiscuous ETS fusion member (49) (Figure 2F).

We observed that fusions of the ETS family members *ERG*, *ETV1*, *ETV4*, and *ETV5* were mutually exclusive, except for 1 sample that harbored fusions of both *ERG* and *ETV1* (Supplemental Figure 2I). In addition, gene fusion events were correlated with higher expression of the corresponding ETS genes they involved (Wilcoxon’s rank-sum tests, *P <* 0.05 for all genes; Figure 2E). In the 38 cases that did not show any evidence for an ETS fusion, we noted that presence of high-level expression (*z* score > 1) of the ETS genes *ERG*, *ETV1*, *ETV4*, and *ETV5* was also mutually exclusive (Fisher’s exact test, *P =* 0.480 for *ETV4*, *P =* 0.363 for *ETV5*; Supplemental Figure 2I). These may represent cases of missed fusion calls, or cases in which ETS family members are transcriptionally activated through non-genetic mechanisms.

Interestingly, we also observed 20 cases (14.0% of 143 cases) involving fusions between the ETS family member *ELK4* and its upstream gene *SLC45A3*. While the *ELK4* locus was an SRB in our analysis (Figure 2A and Supplemental Figure 2I), manual inspection of individual samples revealed evidence for a genomic event capable of producing an *ELK4* fusion in only 1 of 20 cases (Supplemental Figure 2I and data not shown). In contrast, 19 other cases showed *ELK4* fusions on RNA-Seq alone, consistent with a mechanism of *cis*-splicing or transcriptional read-through events that may perhaps be induced by local genomic alterations (50–52) (Supplemental Table 2D). Importantly, although *ELK4* fusions were significantly correlated with higher expression of *ELK4* (Wilcoxon’s rank-sum test, *P =* 7.91 × 10^–5^; Supplemental Figure 2I), these events were not mutually exclusive with fusions of other ETS family members (Fisher’s exact test, *P =* 0.472). Thus, the functional consequences of these *ELK4* fusions and whether they contribute to prostate cancer pathogenesis in a manner similar to that of other ETS fusions remain to be determined.

### Classes of rearrangements driving AR signaling in mCRPC.

**Figure 3:**
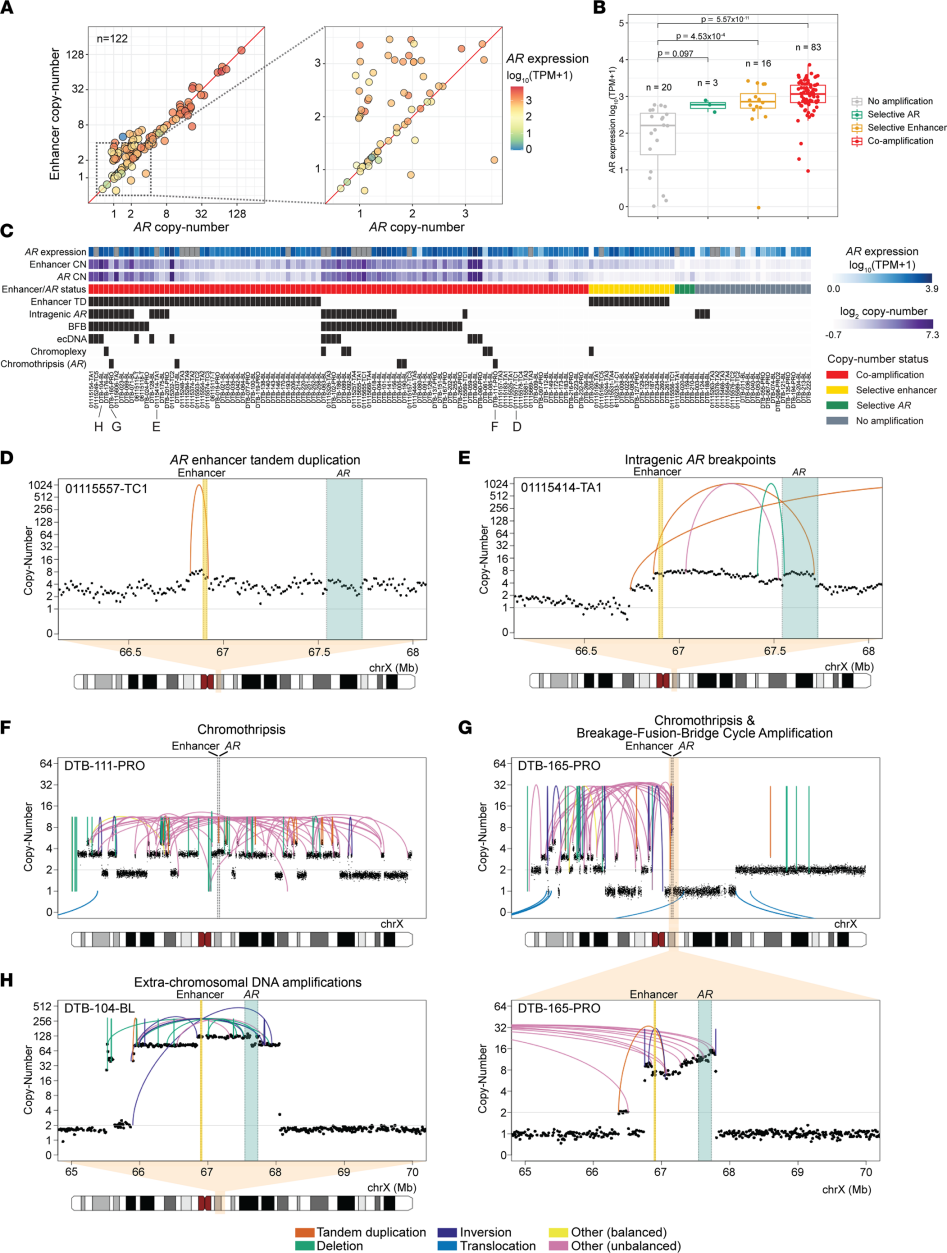
Modes of *AR* activation in mCRPC. (**A**) Copy number of *AR* gene and its enhancer (~624 kb upstream) for mCRPC cohort samples after adjustment by tumor purity and sample ploidy normalization. Data shown for samples with available *AR* gene expression data. Left: Copy number of *AR* and its enhancer is shown in log_2_ scale, colored based on *AR* gene expression level (transcripts per million [TPM]). Right: Excerpt of figure highlighting *AR* expression for samples with lower copy number values. (**B**) *AR* expression for *AR* locus copy number status for 122 samples with available *AR* gene expression data. ANCOVA test was performed to account for tumor purity and ploidy as covariates. Tukey’s HSD *P* values for pairwise comparisons between groups with *AR* locus amplification status and groups with no amplification. (**C**) Patterns of rearrangements involving the *AR* locus in 143 mCRPC samples. Presence of specific alteration events and complex rearrangements (black) was predicted automatically and manually curated. *AR* gene expression shown (blue shades) for the same samples in **B**; samples with no available expression data are indicated in gray. (**D**–**H**) Representative examples of each category. Complex and simple rearrangement patterns involving the *AR* locus, including focal duplication events on *AR* enhancer (**D**), intragenic amplification event leading to a breakpoint within *AR* between exons 4 and 5 (**E**), chromosome-level chromothripsis events involving *AR* and enhancer (**F**), arm-level chromothripsis coinciding with *AR* amplification by break-fusion-break cycle (**G**), and extrachromosomal DNA amplicon including *AR* and enhancer (**H**). *AR* gene boundary (green) and its enhancer (yellow) are shown. Concave arcs, intrachromosomal SV events; convex arcs, interchromosomal SV events. Copy number values represent 10 kb bins and have been tumor purity corrected.

Genomic alterations involving the *AR* locus play an important role in sustaining *AR* signaling in mCRPC (13, 15, 26, 53). We sought to catalog the spectrum of diverse structural mechanisms that underlie *AR* activation in mCRPC, and the relationship between them, in our combined mCRPC cohort. To understand the relationship between different modes of somatic *AR* activation, we first determined copy number at the *AR* gene body and its upstream enhancer and categorized samples into distinct groups of (a) coamplification (*n =* 99, 69.2% of 143 cases); (b) selective *AR* gene body amplification (*n =* 4, 2.8% of 143 cases); (c) selective *AR* enhancer gains (*n =* 17, 11.9% of 143 cases); and (d) lack of amplification for both (*n =* 23, 16.0% of 143 cases) (Figure 3, A–C, and Supplemental Table 3). For the 125 samples with expression data available, we observed that *AR* gene expression was higher in the coamplification and selective enhancer categories compared with samples with no amplification, after accounting for tumor purity and ploidy (ANCOVA/Tukey’s honestly significant difference [HSD] *P* values 5.6 × 10^–11^ and 4.5 × 10^–4^, respectively), but not for selective *AR* status (ANCOVA, *P =* 0.098) (Figure 3B). Interestingly, we observed that samples with selective enhancer duplication exhibited similar *AR* expression levels to samples with coamplification (ANCOVA, *P =* 0.31), even though enhancer duplications involved lower copy number gains (mean 2.73, range 1.97–5.02) compared with coamplified samples (mean 12.87, range 1.55–150.57) (Figure 3A). This is consistent with previous results (15, 28) and suggests a mechanism whereby *AR* expression levels are increased through even modest genomic expansion of enhancer elements.

We then systematically and manually curated the diverse mechanisms of rearrangements activating *AR* signaling by analyzing patterns of SVs at the *AR* locus (Figure 3C and Supplemental Table 3). We observed a total of 62 samples (43.4% of 143 samples) with tandem duplication SV events that spanned the enhancer with breakpoints located within 1 Mb, including 16 cases (11.2% of 143 samples) with selective enhancer copy number amplification status (Figure 3D). Thirty-two samples (22.4% of 143 samples) harbored intragenic rearrangements within *AR*, which may have implications for the production of truncated, constitutively active *AR* splice variants (31). For example, in case 01115414-TA1, we observed a tandem duplication breakpoint selectively amplifying exons 1–4 of *AR*, but not exons 5–8 of *AR*, which includes the ligand-binding domain; such an event could promote selective expression of a constitutively active truncated *AR* variant, although RNA-Seq data were not available on this sample (Figure 3E). In another case, DTB-124-BL, our reanalysis confirmed that a focal deletion involving exons 1–4 resulted in the expression of truncated *AR* variants, as previously described (28) (Supplemental Figure 3C). Interestingly, of the 21 samples with selective *AR* enhancer or selective *AR* gene body copy number gain, none harbored intragenic SV events in *AR*.

We also examined the landscape of complex rearrangement mechanisms involving *AR*; these mechanisms involve multiple SV events and copy number patterns, including chromothripsis, extrachromosomal DNA (ecDNA), chromoplexy, and breakage-fusion-bridge cycle (BFB). Chromothripsis of a region or the entire X chromosome involving the *AR* locus was detected in 5 samples, all of which had coamplification of *AR* and enhancer, suggesting that, following repair after catastrophic DNA shattering, the *AR* locus was retained or further amplified (Figure 3, F and G). Thirteen samples (9.1% of 143 samples) showed very high levels of *AR* and enhancer copy number, suggesting the possibility of their presence on extrachromosomal elements (ecDNA; Figure 3H). In 40 samples (28.0% of 143 samples), the most frequent complex rearrangement mechanism, BFB, led to *AR* locus amplification, including instances following chromothripsis (14, 54) (Figure 3G). Overall, we noted that complex rearrangement events, which frequently co-occurred, were significantly enriched in samples with coamplification of *AR* and enhancer compared with those with selective enhancer copy number gain status (Fisher’s exact test, *P =* 1.52 × 10^–4^).

### Distinct signatures of structural rearrangement patterns in mCRPC.

To systematically characterize genome-wide structural rearrangement patterns in mCRPC, we performed rearrangement signature analysis using SV breakpoint features, non-negative matrix factorization, and known reference signatures (12, 55). First, we derived signatures de novo, which identified 8 signatures: 6 that matched reference signatures (RefSigs) also observed in localized prostate cancer (>0.91 cosine similarity), 1 that matched an ovarian cancer RefSig, RefSig.R14, associated with large-segment (100 kb to 10 Mb) TDP (0.96 cosine similarity), and 1 that was likely an artifact specific to linked-read sequencing (Supplemental Figure 4, A–C, and Supplemental Table 4, A and B). Therefore, we excluded the linked-read data and focused on standard WGS data from 101 mCRPC cases for further SV signature analysis. We fit standard WGS samples to the 9 known RefSigs from localized prostate cancer (R1–4, R6a–b, R8–9, R15) and the one (R14) from ovarian cancer (Supplemental Figure 4A and Supplemental Table 4C). Overall, 8 of these 10 RefSigs were detected across our cohort (R1–2, R4, R6a–b, R9, R14–15). Notably absent in mCRPC were RefSig.R8 (short, 1–10 kb inversions) and RefSig.R3, which is associated with germline *BRCA1* mutations and short (1–100 kb) tandem duplications (11, 12, 55, 56) (Supplemental Figure 4D). We also observed increased prevalence of some signatures in mCRPC compared with localized disease, including RefSig.R2 (large SV classes, abundant translocations; 97% vs. 60%), RefSig.R4 (clustered translocation events; 37% vs. 27%), and RefSig.R15 (large deletions and inversions; 48% vs. 37%) (Supplemental Figure 4D).

**Figure 4:**
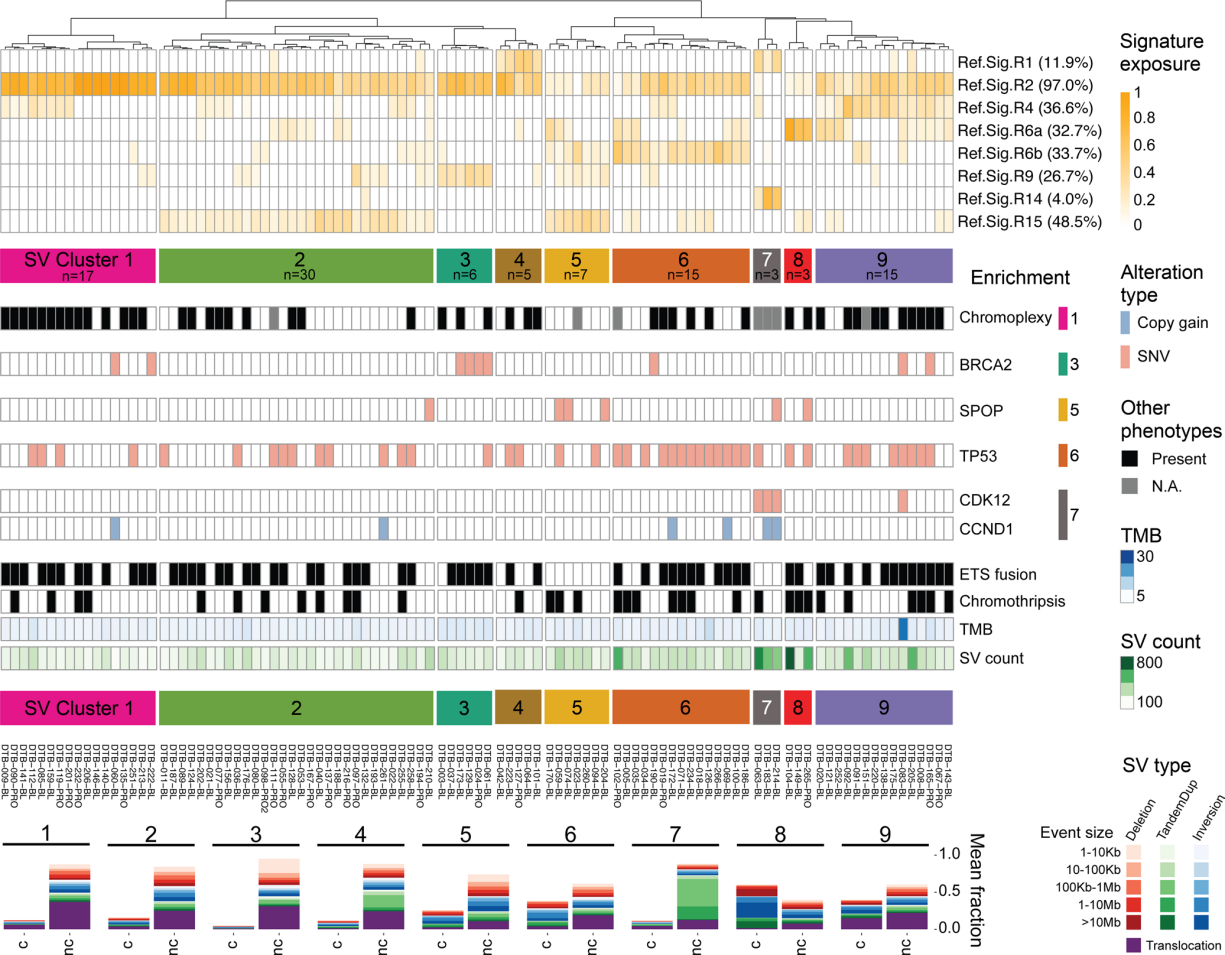
Clustering of mCRPC SV signatures. SV signature analysis and hierarchical clustering identify 9 distinct molecular groups in the mCRPC cohort of 101 samples sequenced with standard short reads. Top: Dendrogram of the clustering of SV signature exposure. The prevalence of each signature was computed based on having ≥0.05 exposure (proportion of SVs). Middle: Enrichment of altered prostate cancer drivers. Enriched alterations in clusters 1, 3, 5, 6, and 7 are shown based on statistical significance by χ^2^ test. Bottom: Composition of SV types and sizes for each SV cluster, separated by non-clustered (nc) and clustered (c) SV events. The number of samples per cluster is indicated in the corresponding cluster label.

To investigate whether molecular subtypes in mCRPC can be grouped based on SV patterns, we applied hierarchical clustering on the exposure of the 8 fitted signatures and identified 9 distinct SV clusters (Figure 4 and Supplemental Table 4C). We observed that samples in SV cluster 1 were composed of non-clustered translocation events and were significantly enriched for the presence of chromoplexy (χ^2^ test, FDR corrected, *q* = 0.12). SV cluster 3 was characterized by many short deletions and was significantly enriched for *BRCA2* mutations (*q* = 5.01 × 10^–4^). SV cluster 5 was significantly enriched for *SPOP* mutations (*q* = 0.02), with no instances of ETS gene family fusion (*q* = 0.06), consistent with previous reports (57). SV cluster 6 had the highest prevalence of *TP53* mutation (*q* = 0.02), while SV cluster 7 samples harbored the TDP associated with *CDK12* inactivation (*q* = 3.52 × 10^–11^) as well as enrichment for *CCND1* gains (*q* = 0.02), consistent with previous reports (35, 58). The remaining clusters did not have enrichment for any alterations in known driver genes; however, distinct SV patterns were still evident in SV clusters 4 (non-clustered tandem duplications), 8, and 9 (increased clustered SV events of various classes).

While SV clusters 3, 5, and 6 had significant enrichment of mutations in *BRCA2*, *SPOP*, and *TP53*, respectively, not all samples within each cluster harbored these mutations. Intriguingly, we further noted that clinical outcomes showed significantly better stratification using SV clusters 3, 5, and 6 for outcome stratification compared with using the associated mutation status itself (Supplemental Figure 4, D and E). Specifically, SV cluster 5 had significantly better overall survival than SV clusters 3 and 6 (log-rank test, *P* = 0.01), while the sample group with *SPOP* mutations did not have significantly greater survival compared with the sample groups with *BRCA2* and *TP53* mutations (log-rank test, *P* = 0.45) in this cohort. However, 2 important limitations of these analyses are (a) that certain SV signatures may be platform dependent (as observed for linked-read WGS), and (b) the absence of a large WGS validation cohort in which to verify cluster abundance as well as association with clinical outcomes. Nonetheless, together, these results indicate that the analysis of genome-wide patterns of rearrangements may provide a way to further refine molecular subtypes in mCRPC.

## Discussion

We present a large-scale and comprehensive integrative genomic analysis of both localized prostate cancer and mCRPC, with a focus on how structural variation drives each of these clinically distinct disease states. The size of our cohort as well as our harmonized analysis pipeline enables a sharper view of the genetic alterations that drive prostate cancer across its natural history as compared with prior studies, which have either involved smaller cohorts or been limited to a single disease state (9, 13, 15, 59).

Our analysis combined WGS data from linked-read and standard short-read sequencing technologies, which demonstrated comparable detection of SVs (Supplemental Table 1M). Notably, the similar SV results between the platforms were achieved with only one-third of the sequencing coverage in the mCRPC samples with linked reads (~34×) compared with those with short reads (~100×). This observation is consistent with the notion that linked reads may provide improved physical genome coverage with less overall sequencing, an advantage of higher–molecular weight DNA in the library (60). However, we also observed an SV signature composed of short tandem duplications, which was a potential linked-read-specific artifact (Supplemental Figure 4). Additional studies directly comparing samples sequenced on both platforms will help to quantify the differences and advantages of the 2 sequencing methods.

In contrast to somatic SNVs/indels and CNAs that occur within coding regions, the functional and clinical significance of alterations within noncoding regions has often been more challenging to interpret, as localized variations in mutability may result in the nomination of certain recurrently mutated sites that do not necessarily drive cancer (11, 12, 44). This issue is even more complex for SVs, in which different classes of SVs spanning the same loci would be predicted to have distinct functional consequences. Our study addresses the former issue by identifying genomic hotspots of structural variation with rigorous correction for covariates including nucleotide composition, replication timing, sensitivity to DNA breaks, repetitive elements, and chromatin state. We address the latter issue by careful curation of SV classes to distinguish those that are likely to be activating versus inactivating (Figure 1B and Figure 3).

Our approach has produced several insights into the recurrent rearrangements that drive prostate cancer. First, several top hotpots of rearrangement genome-wide lie in noncoding regions outside the boundaries of known prostate cancer genes, as previously reported (10, 13, 15). In many cases, such as for *AR*, *MYC*, and *FOXA1*, these hotspots overlap with active chromatin marks and likely represent distal regulatory regions for neighboring prostate cancer genes, as shown by our analyses overlapping SVs with ChIP-Seq on mCRPC specimens (46) (Figure 2). These data are intriguing in light of the observation that a majority of prostate cancer germline susceptibility loci are in noncoding regions (61). Second, the loci altered by rearrangements differ across prostate cancer disease states (Figure 2B). For example, *TMPRSS2-ERG* rearrangements are enriched in localized prostate cancer versus mCRPC, while alterations in *AR*, *FOXA1*, *MYC*, and *LSAMP* are more frequent in mCRPC than in localized disease. Third, certain driver genes are enriched for alteration by SVs as compared with other mutagenic processes. For example, *PTEN* inactivation frequently occurs via gene transecting SV events, while *TP53* inactivation is primarily caused by SNVs (Figure 1C and Supplemental Figure 1).

Our systematic genomic discovery efforts confirm the primacy of *AR* as a target of somatic alteration in hormone-refractory mCRPC (13, 15, 26, 53, 62). We have precisely catalogued the diverse genomic mechanisms leading to *AR* activation across our large aggregate cohort and find that different alteration mechanisms are associated with differing levels of *AR* amplification. Whether the precise mechanism by which *AR* is altered in a given patient is associated with differences in response to *AR* pathway inhibition warrants further investigation in clinically annotated cohorts. High levels of *AR* signaling in mCRPC may also underlie the patterns of structural variation seen in this disease state. Strikingly, we found that *AR* binding sites overlapped several of the top SV hotspots in mCRPC (Figure 2D), consistent with the notion that androgen signaling may induce DNA double-strand breaks that resolve as rearrangements (63).

In addition to alterations in highly validated prostate cancer genes, we identified highly recurrent rearrangements near or involving genes that have not been extensively studied in prostate cancer in multiple cohorts, such as *LSAMP*, *PTPRD*, and *TTC28*. *LSAMP* encodes a cell-surface glycoprotein and has a possible tumor suppressor role in several cancers (40–42); notably, deletions near the *LSAMP* locus have been shown in one report to be enriched in African American men with prostate cancer (64). *PTPRD*, a receptor protein tyrosine kinase, has been previously identified as a target of inactivating alteration in glioblastoma (43). We observed frequent SVs near the *TTC28* locus, which encodes an L1 retrotransposon element, specifically in mCRPC (Figure 1C). L1 retrotranspositions originating from *TTC28* have been reported previously in colorectal cancer (37–39); our results raise the intriguing possibility that they may also be frequent in prostate cancer, and may be activated by the pressure of hormonal therapy. Interestingly, we also observed SRBs near *ELK4* along with a relatively high frequency of *SLC45A3-ELK4* chimeric transcripts, although it was not clear how the rearrangements at this locus produced the chimeric transcripts in most cases. Whether this fusion functions similarly to or in a distinct mode from other ETS fusions is an exciting area for future study.

Our study also extends beyond the analysis of SVs at individual loci to molecularly subclassify prostate cancers based on their genome-wide signatures of structural variation. Sample clustering based on SV signature exposure defines distinct molecular subtypes of prostate cancer and may find utility alongside signatures of single base substitution and copy number to more precisely define tumor subtypes (55, 65–68). In the mCRPC cohort, we identified 9 molecular subtypes based on SV signature, and several clusters had clearly associated genomic alterations including chromoplexy (cluster 1), *BRCA2* alterations (cluster 3), *SPOP* alterations (cluster 5), *TP53* alterations (cluster 6), and *CDK12*/*CCND1* alterations (cluster 7). Notably, unsupervised clustering identified samples with distinctive SV signatures but without detectable associated mutations in genes or pathways that plausibly contribute to those signatures (Figure 4). Moreover, in the data sets analyzed, clinical outcomes were more separated by SV signature cluster than by alterations of the mutations associated with those clusters (Supplemental Figure 4, D and E). Future studies with larger WGS and RNA-Seq cohorts will be required to validate these clusters, to identify their associated alterations and/or transcriptional signatures, and to validate association with clinical outcomes.

In sum, these results highlight the dynamic complexity of rearrangements in prostate cancer across disease states and provide insights into new mechanisms of oncogenesis that can be functionally prioritized in future studies. More broadly, our work underscores the key role of large-scale WGS studies in the derivation of a comprehensive molecular taxonomy of prostate cancer.

## Methods

Supplemental Methods are available online with this article.

### Data and code availability

Whole genome linked-read sequencing data were deposited at the Database of Genotypes and Phenotypes (dbGaP) under accession number phs001577, and access is available upon request. Whole genome short-read sequencing data for 101 mCRPC samples were obtained from dbGaP accession phs001648. Localized prostate cancer structural variant callsets were obtained from International Cancer Genome Consortium (ICGC)/The Cancer Genome Atlas (TCGA) PCAWG (https://dcc.icgc.org/releases/PCAWG/consensus_sv and https://data.mendeley.com/datasets/6gtrrxrn2c/1). All original code was deposited at GitHub and is publicly available as of the date of publication (https://github.com/GavinHaLab/crpc-sv-pattern-study with commit ID 73096df). Any additional information required to reanalyze the data reported in this paper is available from co–corresponding author GH upon request.

### Statistics

#### Association of AR locus amplification status and AR expression.

ANCOVA test was used to test whether different patterns of *AR* amplification have an impact on *AR* expression. Batch-corrected log_10_(transcripts per million + 1) values using ComBat from the sva R package (v3.34.0) were used for *AR* expression level. We fit the ANCOVA model using *AR* expression as the response variable, *AR* amplification status as the predictor variable, and ploidy and purity as covariates. The function Anova in the car package (v3.0-5) was used with type III sum of squares for the model. Post hoc analysis was performed to determine the specific differences among 4 different *AR* amplification statuses. The function glht was used within the multcomp package (v1.4-11) in R to perform Tukey’s test for multiple comparisons.

#### Enrichment of alterations in SV clusters.

All 9 identified SV clusters were analyzed for enrichment of alterations. To make the analysis unbiased by SV signature, we limited our search to alteration types that were orthogonal to rearrangements, which include SNV, copy number gain, and copy number loss. We performed hypothesis testing on each driver-alteration pair, and also on chromoplexy and chromothripsis. For each SV cluster, a χ^2^ test was performed for each driver gene alteration status, with samples within group being tested against samples belonging to all 8 other SV clusters. Multiple testing adjustment based on Benjamini-Hochberg FDR was performed to compute *q* values. Alteration categories with *q* values less than 0.25 were determined as enriched in the corresponding SV cluster.

#### Survival analysis.

Survival data were obtained from ref. 69. Survival analyses were conducted using the Kaplan-Meier method with log-rank testing for significance. The function survfit from survival R package was used to perform the analysis.

### Study approval

For tumor biopsies profiled via linked-read sequencing, samples were collected from individuals with mCRPC who provided informed consent on IRB-reviewed protocols, as previously described (15). Uniformly reanalyzed data were generated as described in the respective studies (9, 13, 18).

## Author contributions

Conceptualization was contributed by MET, MM, SRV, and GH. Methodology was contributed by MZ, MK, SRV, and GH. Software: MZ, MK, ACHH, and GH. Formal analysis was contributed by MZ, MK, ACHH, KL, YL, MLR, WWH, JCZ, SRV, and GH. Data curation was contributed by MZ, MK, ACHH, ZZ, SRV, and GH. Writing of the original draft was contributed by MZ, MM, GH, and SRV. Review and editing of the manuscript were contributed by MZ, RB, EMVA, ADC, PSN, MLF, MET, MM, GH, and SRV. Visualization was contributed by MZ, MK, ACHH, SRV, and GH. Supervision was contributed by MET, MM, SRV, and GH. Funding acquisition was contributed by SRV, GH, and MM.

## Supplementary Material

Supplemental data

Supplemental table 1

Supplemental table 2

Supplemental table 3

Supplemental table 4

## Version 1 08/09/2022

In-Press Preview

## Version 2 09/08/2022

Electronic publication
