# Supplementary material for: Patterns of structural variation define prostate cancer across disease states
Source: JCI Insight. 2022 Sep 8;7(17):e161370. doi: 10.1172/jci.insight.161370 (PMC9536266; doi:10.1172/jci.insight.161370)
Supplement: Supplemental data [file jciinsight-7-161370-s216.pdf]

Patterns of Structural Variation Define Prostate Cancer Across Disease States

Meng Zhou[1,2] *, Minjeong Ko[4] *, Anna C. Hoge[4], Kelsey Luu[4], Yuzhen Liu[4], Magdalena L. Russell[4], William W. Hannon[4], Zhenwei Zhang[1,5], Jian Carrot-Zhang[1,2,3], Rameen Beroukhim[1,2], Eliezer M. Van Allen[1,2,6], Atish D. Choudhury[1,3], Peter S. Nelson[4,7], Matthew L. Freedman[1,3,8], Mary-Ellen Taplin[1,3] †, Matthew Meyerson[1,2,3] †, Srinivas R. Viswanathan[1,2,3] †, Gavin Ha[4,7] †

Supplementary Materials

Supplementary Methods

Sequence data processing for linked-read genome sequencing data

Data processing of the linked-read genome sequencing data include high molecular weight DNA preparation and sequencing library construction followed protocols as previously described (1). DNA was extracted from tumor samples using the MagAttract HMW DNA Kit (QIAGEN), and then quantified using Quant-it Picogreen assay kit (Thermo Fisher) on a Varioskan Flash Microplate Reader (Thermo Fisher). For germline samples, pre-extracted DNA was size-selected on the PippinHT platform (Sage Science) and then quantified using the Quant-it Picogreen assay kit (Thermo Fisher) on a Varioskan Flash Microplate Reader (Thermo Fisher). Libraries were constructed using the 10X Chromium protocol (10X Genomics), with the fragment sizes determined using the DNA 1000 Kit and 2100 BioAnalyzer (Agilent Technologies) and quantified using qPCR (KAPA Library Quantification Kit, Kapa Biosystems). WGS libraries were sequenced using the Illumina HiSeqX platform. The Long Ranger v2.2.2 pipeline (10X Genomics) was used for aligning sequence reads to the human genome hg38 (GRCh38).

Samples were excluded from the analysis based on having tumor purity less than 15% estimated by TitanCNA or based on cross-individual contamination indicated by SNP fingerprinting. Based the 15% tumor purity threshold, an average coverage of ~34x will provide 47% theoretical power to detect a heterozygous mutation. Power is computed for detecting a minimum of 3 supporting reads or mutant reads using the following binomial exact test,

$$p(X \geq 3) = 1 - [Bin(0|N,\mu) + Bin(1|N,\mu) + Bin(2|N,\mu)],$$

where

$$\mu = \frac{\alpha M}{\alpha c_T + 2(1-\alpha)}$$

for tumor purity α, read depth N, diploid copy number $c_T = 2$, and multiplicity $M = 1$ for a heterozygous event. However, the 47% power is lower bound estimate as the three lowest purity

samples ranged between 16-17% and had coverages ~39x, which provided >62% power (Table S1B). In total, 17 samples with linked-read data were excluded (**Table S1J**). For short read data in the mCRPC cohort, we retained all 101 samples because the average sequencing coverage was ~100x, which provided 63.5% power for the sample with the lowest purity (6.5% for DTB-193-BL, 113x coverage) and 93.9% power for the second lowest purity (11.82% for DTB-018-BL, 108x coverage) (Table S1B).

List of known prostate cancer driver genes

For analyses limited to established prostate cancer driver genes, a curated list of 159 known prostate cancer driver genes was assembled from several prior studies (1–4). The list of genes is provided in **Table S1**.

Somatic mutation analysis

Somatic mutation detection

Somatic mutation calls for samples based on linked-read sequencing were generated by Mutect2 from the Genome Analysis Toolkit (GATK) (5). Default parameters were used on individual pairs of tumor and normal samples following the standard GATK pipeline. A panel of normals based on all normal samples was used to filter out germline variants. The SNV calls were further processed using the modified version of LoLoPicker (6) as described previously (1). The panel of normals for LoLoPicker was generated from 52 normal samples based on linked-read sequencing. The final SNV call set was composed of the common variants called by both Mutect2 and LoLoPicker. Somatic indels for linked-read samples were called by Strelka (7). All parameters were default except the following modifications: sindelNoise = 0.000001, minTier1Mapq = 20. Somatic mutation calls for the 101 WGS samples based on short-read sequencing including SNV and indels based on Strelka were obtained from a prior study (4). All variants were further annotated using annovar with “table_annovar.pl” to functionally annotate genetic variants. The parameter -

neargene was set to 5000 to define the promoter region as 5 kb upstream of the transcription start site of a protein coding gene.

Analysis of significantly mutated genes

R package dndscv (8) was used to identify significantly mutated genes. For driver discovery on GRCh38, a precomputed database corresponding to human genome GRCh38.p12 was downloaded and used as the reference database. A global q-value ≤ 0.1 was applied to identify statistically significant (novel) driver genes. To reduce false positives and increase the signal to noise ratio, we only considered mutations in Cancer Gene Census genes (v81) (9).

Copy-number analysis of linked-read WGS and short-read WGS data

Copy-number calls

The ploidy and purity corrected copy-number of all mCRPC samples in this study was analyzed by TitanCNA (10) and ichorCNA (11), with different pipeline settings. For WCDT samples, the snakemake workflow for Illumina sequencing was applied with the following parameters modified: ichorCNA_normal: c(0.25, 0.5, 0.75); ichorCNA_ploidy: c(2,3,4); ichorCNA_includeHOMD: TRUE; ichorCNA_minMapScore: 0.75; ichorCNA_maxFracGenomeSubclone: 0.5; ichorCNA_maxFracCNASubclone: 0.7; TitanCNA_maxNumClonalClusters: 3; TitanCNA_maxPloidy: 4. The workflow is available at https://github.com/GavinHaLab/TitanCNA_SV_WGS.

For linked-read data samples, a Snakemake workflow for 10X Genomics whole genome sequencing data was used with the following parameters modified: TitanCNA_maxNumClonalClusters: 3; TitanCNA_maxPloidy: 4. TitanCNA solutions were generated for number of clonal clusters from 1 to 3 and ploidy initializations from 2 to 4. Optimal solutions were selected as described, with manual inspection to confirm tumor ploidy and clonal cluster selection (1); solutions are provided in **Table S1J**. The workflow can be accessed at

https://github.com/GavinHaLab/TitanCNA_10X_snakemake. The final copy-number call-set is included in **Table S1I**.

Recurrent somatic copy-number alteration

GISTIC 2.0 was used to detect regions with recurrent CNA in mCRPC samples. For input, all copy numbers (logR_Copy_Number from TITAN output) were converted to log2 copy ratio using the median logR copy number from genome-wide (separately for autosomes and X chromosome) as denominator. We set corrected logR copy number to -1.5 for segments where corrected log R copy number below -1.5 and set values to 0 if copy neutral. GISTIC2.0 was run with the following parameters: td 0.5; ta 0.1; genegistic 0; maxseg 5000; js 4; cap 1.5; broad 1; brlen 0.75; conf 0.99; qvt 0.25; armpeel 1; rx 0; gcm mean; do_gene_gistic 1; savegene 1; scent median. Wide peaks detected by GISTIC2 were re-annotated based on overlapping genomic coordinates, using prostate cancer driver genes.

Structural variant analysis

Structural variant detection in linked-read and short-read whole genome sequencing data

For each tumor-normal pair of samples with linked-read genome sequencing data, three variant callers were used to detect structural variants: SvABA (12), GROC-SVS (13), Long Ranger version 2.2.2 (https://support.10xgenomics.com/genome-exome/software/pipelines/latest/using/wgs).

The SvABA analysis was performed using default tumor-normal paired settings. Re-analysis of low confidence (based on evidence from discordant and split reads) events filtered by SvABA was performed to 'rescue' SVs using linked-read barcode overlap between pairs of breakpoints within a given SV event, as previously described (1). Only SV events having span of 1.5 times the mean molecule length in the library were considered for rescue. We further rescued low confidence intra-chromosomal SV events with span > 50 kb filtered by SvABA if at least one of the breakpoint

pair was within 100 kb of a CNA boundary or (2) if both breakpoints were each within 1 Mb of the boundaries for the overlapping CNA event and the length of the SV overlaps this CNA event by > 75%. Inter-chromosomal translocation SV events filtered by SvABA are rescued if both breakpoints were within 100 kb of CNA boundaries.

GROC-SVS analysis was performed using two-sample (tumor-normal paired) mode or three-sample (pre-treatment, post-progression, normal) mode when applicable. SV events were retained if all following conditions were satisfied: (1) $p < 1x10^{-10}$, (2) minimum barcode overlap ≥ 2 on the same haplotype, (3) no more than 1 barcode overlap between different haplotypes, (4) FILTER value reported by the software was within this set {"PASS", "NOLONGFRAGS", "NEARBYSNVS", or "NEARBYSNVS; NOLONGFRAGS"}, and (5) classified as somatic.

Long Ranger analysis generated SV calls for tumor and normal samples, independently. For each tumor-normal pair, both large SVs ("large_sv_calls.bedpe") and deletions ("dels.vcf") were combined for individual samples. Somatic tumor SVs were determined as events that were not found in the matched normal sample based on the left breakpoints in tumor and normal being within 1 kb and the right breakpoints in tumor and normal samples being within 1 kb. Only SV events with FILTER values within this set {"PASS", "LOCAL_ASM", "SV", "CNV, SV"} and intra-chromosomal events with span ≥ 100 kb were considered. SV events were only retained if both breakpoints of an SV event were within 500 kb the boundaries of an overlapping CNA event and the length of SV overlaps this CNA event by > 75%.

SV events from these three callers were then combined by taking the union of the filtered events from. Intersecting events between 2 or more call-sets were determined if both breakpoints of one event were located within 5 kb from both breakpoints of the event detected by the other tool. Then the details of this event were retained based the priority ordered by SvABA, GROC-SVS, Long Ranger. Long Ranger SV events were further filtered out if they were not intersecting events

detected by at least one other tool. SV events with span less than 1 kb were excluded from downstream analyses.

An SV panel of normals (PoN) was generated using germline events from SvABA and Long Ranger calls. There are two components to this panel: (1) frequency of germline events at exact breakpoint locations (SVpon.bkpt) and (2) frequency of germline event breakpoint overlapping within tiled windows of 1 kb (SVpon.blackListBins). The PoN was used to filter events in the combined SV call-set when an SV has at least one breakpoint with SVpon.bkpt ≥ 2 and overlapping bin with SVpon.blackListBins ≥ 100.

The workflow for SV analysis from linked-read sequencing data can be accessed at https://github.com/GavinHaLab/SV_10X_analysis. Manual curation of filtered SV events in the *AR* locus was performed and rescued events were labeled "Manual". The final SV call-set is included in **Table S1K**.

For samples based on short-read WGS, SvABA was used in tumor-normal paired mode for SV detection with default parameters. Intra-chromosomal SV events with span > 1 kb were retained. The SvABA workflow can be accessed at https://github.com/GavinHaLab/TitanCNA_SV_WGS

Classification of structural variants in mCRPC

SV types were annotated based on orientations of breakpoints and bin-level copy-number around breakpoints. The orientation of one breakpoint was defined based on the fragment of DNA molecule being connected to the altered molecule. If the connected fragment was to the 5'-end of the breakpoint, *i.e.*, "upstream" or "left" to the breakpoint, then the orientation was annotated as forward or "+"; on the contrary, if the connected fragment was located to the 3'-end of the breakpoint, the orientation was annotated as reverse or "-". The copy-number near each breakpoint was evaluated using 10 kb bins. For one SV event, copy-number values of the bins located to the upstream and downstream of breakpoint 1 were denoted as c_1^{up} and c_1^{down}, respectively; similarly, the copy-number values for breakpoint 2 were denoted as c_2^{up} and c_2^{down}.

In addition, then mean copy-number c^{mean} of the 10 kb bins between the two breakpoints of one SV event and the number of bins s were also considered during SV classification. Intra-chromosomal SV events, *i.e.*, both breakpoints were located on the same chromosome, were classified to the list of SV types below following the corresponding classification criteria.

- Deletion. Events having the orientation combination (reverse, forward) and length between 10 kb and 1 Mb were classified as deletions. The copy-number values of breakpoints should satisfy $c_1^{up} > c_1^{down}$ or $c_2^{up} < c_2^{down}$, and $c_1^{up} > c^{mean}$ or $c_2^{down} > c^{mean}$, and $s \leq 5$. In addition, events overlapping copy-number deletion or LOH segments were also considered as deletions.
- Tandem duplication. Events having the orientation combination (forward, reverse) and length between 10 kb and 1 Mb were classified as tandem duplications. The copy-number values of breakpoints should satisfy $c_1^{up} < c_1^{down}$ or $c_2^{up} > c_2^{down}$, and $c_1^{up} < c^{mean}$ or $c_2^{down} < c^{mean}$, and $s \leq 5$. In addition, events overlapping copy-number gain or copy neutral LOH segments were also considered as tandem duplications.
- Inversion. Events having the orientation combination (forward, forward) or (reverse, reverse) and length between 10 kb and 5 Mb were classified as inversions. Furthermore, inversion events shorter than 30 kb with unequal copy-numbers around either breakpoint were classified as fold-back inversions.
- Balanced rearrangement (balanced). Events having the orientation combination same to inversion (forward, forward) or (reverse, reverse), but length larger than 5 Mb were classified as balanced events. The copy-number values of breakpoints should satisfy $c_1^{up} = c_1^{down}$ and $c_2^{up} = c_2^{down}$, or $c_1^{up} = c^{mean}$ and $c_2^{down} = c^{mean}$.
- Unbalanced rearrangement (unbalanced). Intra-chromosomal events which did not fulfill any of the above criteria and having length larger than 10 kb were classified as unbalanced events.

- All SV events with two breakpoints located on different chromosomes were classified as translocations.

ICGC/TCGA PCAWG localized prostate cancer structural variants

We obtained localized prostate cancer structural variant calls from ICGC Data Portal release 28 (https://dcc.icgc.org/releases/PCAWG/consensus_sv). In this consensus SV file, each SV event was predicted by at least two variant callers. Samples that were classified as prostate adenocarcinoma (PRAD) and early onset prostate cancer (EOPC) were selected. A total of 278 samples were successfully lifted over to genome build GRCh38. To maximize consistency with mCRPC datasets, we used only the PCAWG consensus SVs that included "SNOWMAN" as one of the tools. Note that "SNOWMAN" was the previous name for SvABA. Intrachromosomal SV events shorter than 10 kb were excluded.

We obtained ICGC early-onset PC (EOPC) and late-onset (LOPC) structural variant calls from Gerhauser et al, 2018 (14) (https://data.mendeley.com/datasets/6gtrrxrn2c/1). This dataset includes a total of 253 samples, consisting of 206 EOPC and 47 LOPC. We used the SV calls generated in the original study using DELLY2 (Rausch et al., 2012) PCAWG analysis workflow (https://github.com/ICGC-TCGA-PanCancer/pcawg_delly_workflow). SV coordinates were lifted over to GRCh38.

Tandem duplicator phenotype

For all samples in the combined cohort, the TDP status was predicted using copy-number and SV by counting the number of copy-number segments overlapping with tandem duplication SV events, *i.e.,* gain segments. A sample was considered as TDP if it has more than 300, or 90 gain segments for samples based on linked-read sequencing and short-read sequencing, respectively. The number of segments with gain and median length SV are reported in **Table S1L**.

Chromothripsis analysis

Chromothripsis events were detected by ShatterSeek R package (15). Structural variants calls by SvABA and copy-number calls by TitanCNA were used as input data (excluding Y chromosome). In the input, consecutive segments were joined as one if they had the same copy-number value and centromere regions were filtered out.

Manual inspection was performed for reported chromothripsis-like events after adapting criteria thresholds. For samples based on short-read sequencing, confidence classification criteria were refined from the ShatterSeek documentation. Following criteria were used for high confidence calls: total number of intra-chromosomal structural variants events involved in the event ≥ 10; max number of oscillating CN segments (two states) ≥ 10; satisfying either the chromosomal enrichment or the exponential distribution of breakpoints test ($p \leq 0.05$). For samples based on linked-read sequencing, we filtered these calls based on a weighted score that is primarily determined by the number of SVs in a cluster, with less weight given to CN oscillations. In this analysis, events with a score over 0.8 were considered as high confidence and all other events were excluded. The score is defined based on the following terms (ranges from 0 to 1).

- Weight 0.6 if total number of intra-chromosomal structural variants events involved in the event ≥ 10.
- Weight 0.2 for max number of oscillating CN segments (two states) ≥ 7 or max number of oscillating CN segments (three states) ≥ 14.
- Weight 0.1 for passing chromosomal enrichment test by ShatterSeek.
- Weight 0.1 for passing exponential distribution of breakpoints test.

Chromoplexy analysis

ChainFinder was used to detect chromoplexy events (16). Ten samples that were considered as TDP (01115374-TA2, 01115202-TC2, 01115248-TA3, 01115503-TC2, 01115257-TA4,

01115284-TA9, 01115414-TA1, DTB-063-BL, DTB-183-BL, DTB-214-BL) were excluded from this analysis. In addition, four samples that were found to cause numeric instabilities of ChainFinder were also excluded (DTB-023-BL, DTB-102-PRO, DTB-111-PRO, DTB-151-BL). The SV calls of remaining samples were further filtered to exclude those that were located within 5 Mb from chromosomal ends or overlapping chromothripsis regions. For copy-number input, segments that were determined as copy neutral by TitanCNA were set to have log copy-ratio of 0. Copy-ratio of the other segments were computed from copy-number values generated by TitanCNA divided by 2 for autosomes or 1 for X chromosome. Log copy-ratio values less than -1.5 were set to -1.5. The output of ChainFinder was used for determining chromoplexy status of individual samples. A chromoplexy event was defined as a chain including at least 5 rearrangement events and involving more than 2 different chromosomes. Samples having at least 2 such events were considered positive for chromoplexy status.

ChIP-seq data analysis

ChIP-seq data used in this study were downloaded from Gene Expression Omnibus (GEO) (17, 18) and the Sequence Read Archive (SRA) (19). Short reads were mapped to the human genome GRCh38 (hg38) using bwa (20). Because read lengths were less than 50bp, the bwa aln command with default parameters was used for mapping. MACS2 (21) was used to identify peaks from mapped ChIP-seq data. For histone modification marks, MACS2 callpeak command was applied with --nomodel --broad –extsize 146. For *CTCF* data, MACS2 callpeak command was used with --nomodel --extsize 200. Below is the list of ChIP-seq datasets involved in this analysis.

- H3K4me3, H3K27me3 and *CTCF* (GSE38685) (22).
- H3K36me3 and H3K9me3 (GSE98732) (23).
- H3K4me1 and H3K27ac (GSE73785) (24).

For *AR* binding site (ARBS), the peak files were downloaded from two different datasets and converted to hg38 coordinates. For primary prostate cancer, ARBS data were downloaded from GSE70079 (Pomerantz et al., 2015). The union of all tumor sample peaks was used. For mCRPC, met-specfic ARBS data were obtained from a previous study (26).

Identification of SRB regions

Masking the human genome based on mappability

The human genome was divided into 100 kb non-overlapping bins for detection of significantly recurrent breakpoint regions (SRB). A low-mappability mask was generated for the hg38 genome to screen out out regions that are difficult for variant calling based on short-read sequencing. We adopted procedures from a previous study (27) to construct a mask corresponding to regions with low mappability in the human genome. Below is a list of masked regions included in the low-mappability mask.

- Composition mask. This set of masked regions includes regions with low sequence complexity detected by mdust, regions with long homopolymers detected by seqtk, satellite regions annotated by RepeatMasker (28), and low complexity regions annotated by RepeatMasker.
- Mappability mask. This mask was based on mappability of *k*-mers in the human genome hg38. The value *k* was set to 75 which is half of the read length of WGS data in this study. Each base in the genome was assigned a mappability level, based on the mapping ambiguity of all 75-mers overlapping this specific base. See below for the list of mappability levels. Regions with mappability level 0 and 1 were included in the low-mappability mask.
    - Level 0: all 75-mers overlapping this base could not be mapped to the genome uniquely.
    - Level 1: more than 50% of overlapping 75-mers are not uniquely mapped.

- Level 2: more than 50% of overlapping 75-mers are uniquely mapped with 1-mismatch hits.
- Level 3: more than 50% of overlapping 75-mers are uniquely mapped without 1-mismatch hits.

In addition, we used GATK CallableLoci to mark regions with high confidence of variant detection based on coverage. Together, the intersection of unmasked regions and callable loci were defined as the eligible territories for SRB detection. The 100 kb bins with less than 75% overlap with eligible territories were excluded from the analysis.

Generating covariates for regression analysis

To accurately model the genomic features of mCRPC, we incorporated the following covariates.

- Nucleotide composition, including GC content, CpG fraction and TpC fraction per 10 kb non-overlapping bin in the genome.
- Replication timing of LNCaP (data obtained from ENCODE under accession ENCFF995YGM, lifted over from hg19 to hg38) (29, 30).
- DNase I hypersensitive sites (data obtained from ENCODE under accession ENCFF434GSJ, lifted over to hg38).
- Repeats annotated by RepeatMasker, including LINE, SINE, LTR, DNA transposon and simple repeats.
- Heterochromatin regions inferred by ChromHMM (31) with the 18-state model parameters from the Roadmap Epigenomics Project (32), based LNCaP ChIP-seq data of H3K4me1, H3K4me3, H3K4ac H3K27me3, H3K36me3 and H3K9me3.
- Common fragile sites downloaded from HGNC biomart (33).

SRB detection

Structural variants from the final call set were used for statistical enrichment of recurrent breakpoints within 100 kb bins using a Gamma-Poisson regression implemented in the package, fish.hook (34). Breakpoints of SVs were treated independently. The Benjamini-Hochberg procedure was used for multiple testing correction and bins with q-value ≤ 0.1 were determined to be significant. The distances of individual known driver genes to those significant bins were evaluated based on the shortest genomic distance between the gene and bin boundaries, regardless of gene orientations.

Many covariates had effect sizes that were statistically significant (p-value < 0.05) in either mCRPC or localized cohorts or both (Table S2C). LINE retrotransposon covariate was the only significant covariate among transposable elements. DNase I hypersensitive site (DHS) covariate was only significant in the mCRPC cohort, which may have been due to the DHS data used for regression belonging to the LNCaP cell line (see Methods). The fragile site covariate was only significant in ICGC EOPC (Gerhauser cohort).

Annotation of gene alteration status

Gene alteration by copy-number

Copy-number segments were excluded if their cellular fraction was lower than 0.8, except for those which were determined as copy neutral or copy-number greater than 4. The gene annotation was based on known protein coding genes from GenCode release 30 (GRCh38.p12) (35). For each gene, its copy-number was assigned to the copy-number value and LOH status of the segment that has the largest overlap with it. The gene-level copy-number was normalized based on ploidy of the corresponding sample, with autosomal genes normalized by the inferred ploidy rounded to nearest integer, and X-linked genes normalized by half such value. Then the copy-number status of each gene was categorized based on the following criteria.

- Amplification. Normalized gene-level copy-number is greater than or equal to 2.5.
- Gain. Normalized gene-level copy-number is between 2 and 2.5.
- Homozygous deletion. Normalized gene-level copy-number is 0.
- Deletion with LOH. Normalized gene-level copy-number is between 0 and 1, and LOH status was found.
- Copy neutral LOH. Normalized gene-level copy-number is 1 and LOH status was found.

Gene alteration by structural variant

Gene coordinates were based on ENSEMBL v33 of hg38 (36). Gene body region of one gene was defined as the widest region of all known isoforms collapsed. Gene flanking region was defined as the corresponding two 1 Mb regions next to the gene body region on 5'-end and 3'-end, respectively.

Gene alteration status by genome rearrangements was defined based on the breakpoints and directions of involving structural variant events. A gene in one WGS sample (gene-sample pair) was considered having gene transecting events if any breakpoints of SV events were located within the gene body region. If the gene transecting status did not apply, then this gene-sample pair was examined for gene flanking status if the breakpoints of any intra-chromosomal SV events, including tandem duplications, deletions, and inversions, were located within the gene flanking regions. Additionally, translocation events including intra-chromosomal balanced and unbalanced events which spanned over 10 Mb, and inter-chromosomal translocation events were considered altering the gene flanking regions if any of their breakpoints was in the gene flanking region, and the direction of the SV was going towards the gene body region. The alteration status of rearrangements for each gene-sample pair was exclusive between gene transecting and gene flanking, with the former being prioritized in report.

AR alteration analysis

Copy-number of the *AR* gene (chrX:67,544,623-67,730,619) and the *AR* enhancer region (chrX:66,895,000-66,910,000) were each computed as the mean corrected total copy-number across the 10 kb bins overlapping each region. The copy-number was further normalized by sample ploidy as previously described. Amplification status of *AR* was determined by comparing the log2 fold-change *FC* of enhancer-level over gene-level copy-number. Four distinct groups were defined based on copy-number and *FC* as below.

- Co-amplification. Ploidy normalized copy-number values of both *AR* gene body and enhancer are greater than 1.5.
- Selective *AR* amplification. *FC* < -log2(1.5) and enhancer copy-number is less than 1.5.
- Selective enhancer copy gain. *FC* > log2(1.5) and *AR* gene body copy-number is less than 1.5.
- Lack of amplification for both. All other cases were considered as no amplification for both regions.

ANCOVA test was used to test if different patterns of *AR* amplification have an impact on *AR* expression. See Statistics section.

Gene expression

TPM values for a subset of the samples based on linked-read sequencing were obtained from cBioportal (37, 38). For samples based on short-read sequencing the TPM values were obtained from a previous study (4).

Gene fusion analysis

Fusion status of the main members of the ETS family, including *ERG*, *ETV1*, *ETV4*, *ETV5* and *ELK4* was analyzed. Determination of gene fusion status was based on both DNA and RNA levels.

For DNA, structural variants transecting gene body regions were used. SV events were considered supporting gene fusion only if they satisfy the following criteria: (1) the breakpoints of this event must be located within the ETS gene and another protein coding gene, respectively; (2) the orientation of the breakpoint located within the ETS gene must be pointing towards the coding sequence of ETS domain. For RNA, arriba was used to detect fusion transcripts from RNA-seq data (39). The fusion status was only confirmed if all following conditions were satisfied: (1) the complete ETS domain was included in the fusion product; (2) detection confidence reported by arriba is “high”; (3) coding sequence in the fusion transcript was in sense orientation and no out-of-frame shifts.

SV signature analysis

Signature extraction and clustering

De novo signature extraction was performed on all SV events called by SvABA of the combined cohort using signature.tools.lib (40) with the recommended settings of 20 bootstraps, 200 repeats, the clustering with matching algorithm, the KLD objective function, and RTOL = 0.001. The exposure of one signature in one sample is defined as the median activity of the signature within the sample across all bootstraps. For clustering, the reference signature exposure values for each sample based on short-read sequencing were normalized such that the sum of exposure values per sample is 1, and the normalized exposure values for each signature were mean-centered across all samples. A Euclidean distance matrix was computed and then samples were clustered with the Ward.D2 algorithm using R’s hclust function. We chose the number of clusters to be k = 9 based on dendrogram using cutree function in R.

SUPPLEMENTAL FIGURES

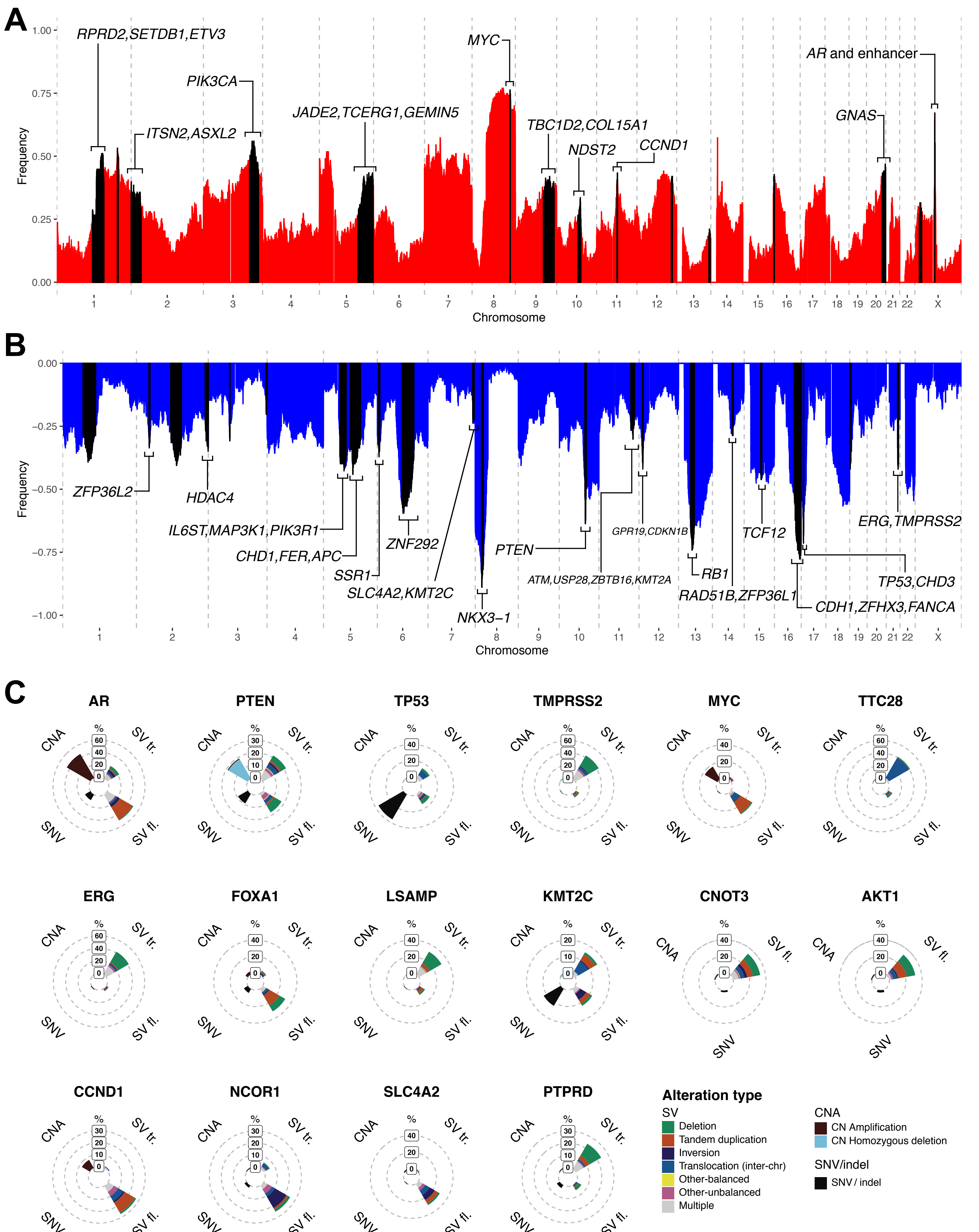
A
RPRD2,SETDB1,ETV3
ITSN2,ASXL2
PIK3CA
JADE2,TCERG1,GEMIN5
MYC
TBC1D2,COL15A1
NDST2
CCND1
GNAS
AR and enhancer
Frequency
Chromosome
B
ZFP36L2
HDAC4
IL6ST,MAP3K1,PIK3R1
CHD1,FER,APC
SSR1
SLC4A2,KMT2C
ZNF292
NKX3−1
PTEN
ATM,USP28,ZBTB16,KMT2A
GPR19,CDKN1B
RB1
RAD51B,ZFP36L1
TCF12
CDH1,ZFHX3,FANCA
TP53,CHD3
ERG,TMPRSS2
C
AR
PTEN
TP53
TMPRSS2
MYC
TTC28
ERG
FOXA1
LSAMP
KMT2C
CNOT3
AKT1
CCND1
NCOR1
SLC4A2
PTPRD
CNA
SV tr.
SV fl.
SNV
%
Alteration type
SV
Deletion
Tandem duplication
Inversion
Translocation (inter-chr)
Other-balanced
Other-unbalanced
Multiple
CNA
CN Amplification
CN Homozygous deletion
SNV/indel
SNV / indel

Figure S1

Figure S1. Recurrent CNA and alteration profiles of most frequently altered genes in mCRPC samples.

(A) Recurrent copy number gain events in the genome of mCRPC samples (n=143). The frequencies of copy number gain are plotted in red according to their genomic coordinates. Regions with significantly recurring CNA are colored in black. Known driver genes that are within those regions are labeled.

(B) Recurrent copy number loss events in the genome of mCRPC samples (n=143). The frequencies of copy number loss are plotted in blue with y-axis inverted.

(C) Alteration profiles of known prostate cancer driver genes. Alterations are categorized into CNA, SNV and SV, with SV being further divided into gene transecting (SV tr.) and gene flanking (SV fl.). The percentages of samples carrying corresponding alterations are shown as stacked bars. All known prostate cancer driver genes were considered and the top 16 genes with overall alteration frequencies above 30% in the mCRPC cohort (n=143) are shown.

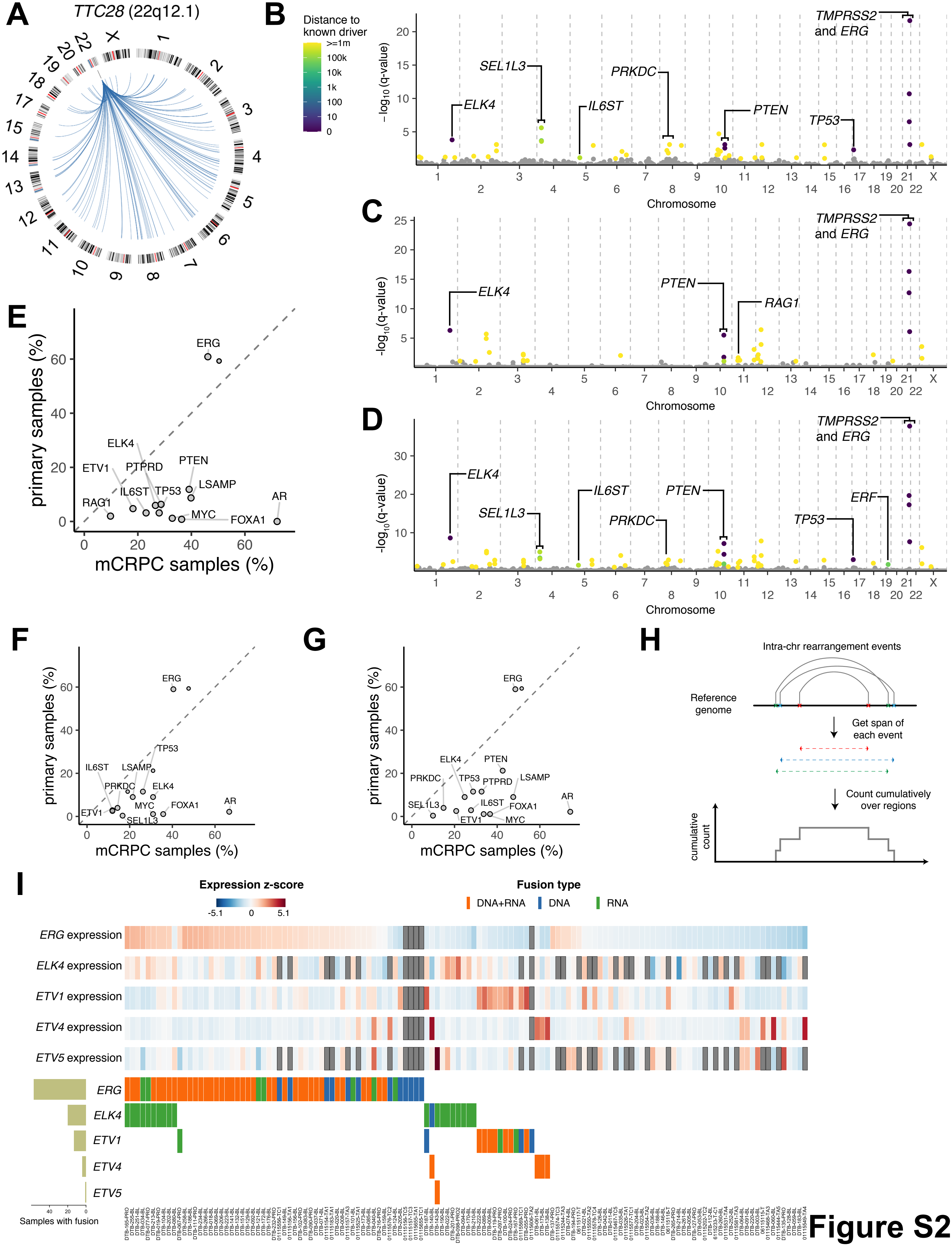
A
TTC28 (22q12.1)
B
Distance to known driver
>=1m
100k
10k
1k
100
10
0
-log10(q-value)
TMPRSS2 and ERG
SEL1L3
PRKDC
ELK4
IL6ST
PTEN
TP53
Chromosome
C
RAG1
D
ERF
E
primary samples (%)
mCRPC samples (%)
ERG
ELK4
ETV1
PTPRD
PTEN
LSAMP
IL6ST
TP53
RAG1
MYC
FOXA1
AR
F
PRKDC
SEL1L3
G
H
Intra-chr rearrangement events
Reference genome
Get span of each event
Count cumulatively over regions
cumulative count
I
Expression z-score
Fusion type
DNA+RNA
DNA
RNA
ERG expression
ELK4 expression
ETV1 expression
ETV4 expression
ETV5 expression
ERG
ELK4
ETV1
ETV4
ETV5
Samples with fusion

Figure S2

Figure S2. Recurrent SV in localized prostate cancer and landscape of ETS fusion in mCRPC.

(A) Translocation events originating from *TTC28* in mCRPC (n=143). In the circos plot, *TTC28* is labeled with a vertical bar at the 22q12.1 locus. Translocation events which have breakpoints located within 5 kB to the 3'-end of the L1 retrotransposon are visualized as blue arcs.

(B) SRBs detected in the cohort of localized prostate cancers from the ICGC Pan-Cancer Analysis Working Group (PCAWG) (n=278). The criteria for coloring and labeling are the same as Figure 2.

(C) SRBs detected in the cohort of localized prostate cancers from ICGC/Gerhauser dataset (n=253). This panel shares legend with panel B.

(D) SRBs detected in localized prostate cancers from the combined cohorts from PCAWG (n=278) and ICGC/Gerhauser dataset (n=253). This panel shares legend with panel B.

(E) Comparison of SV alteration frequency in mCRPC versus primary localized prostate cancer. The union set of genes (n=13) within 1 Mb of SRB hotspot regions in mCRPC (n=143) and localized prostate cancer (ICGC/Gerhauser) dataset (n=253) was included in the comparison. The frequencies represent total gene transecting and flanking SV events. All labeled genes were significantly enriched in either mCRPC or primary localized tumors (Fisher's test, p-value < 0.05).

(F) Comparison of SV alteration frequency in mCRPC samples based on linked-read WGS (n=42) versus primary localized prostate cancer from the PCAWG dataset (n=278). All labeled genes were significantly enriched in either mCRPC or primary localized tumors (Fisher's test, p-value < 0.05).

(G) Comparison of SV alteration frequency in mCRPC samples based on short-read WGS (n=101) versus primary localized prostate cancer from the PCAWG dataset (n=278). All labeled genes were significantly enriched in either mCRPC or primary localized tumors (Fisher's test, p-value < 0.05).

(H) Schematics of cumulative counts from intra-chromosomal SV events. Individual SV events are indicated by a grey arc, and colored crosses correspond to breakpoints of each event.

(I) Expression and fusion status for main genes of the ETS family. The expression values were normalized from TPM to z-score within each gene. Grey boxes indicate expression data are not available. For fusion status, color indicates the data type which was used to call fusion.

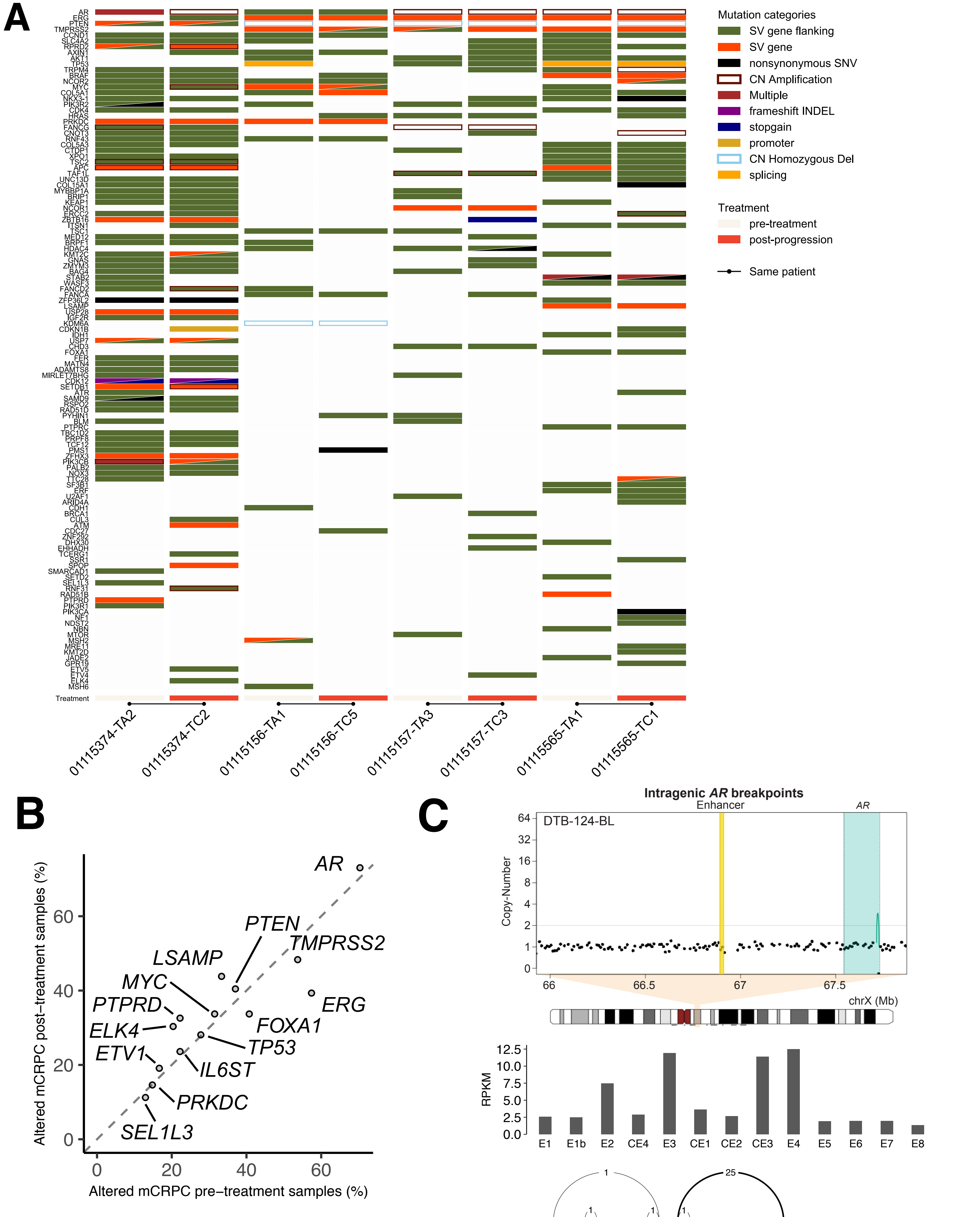
A
AR
ERG
PTEN
TMPRSS2
CCND1
SLC4A2
RPRD2
AXIN1
AKT1
TP53
TRPM4
BRAF
NCOR2
MYC
COL5A1
NKX3-1
PIK3R2
CDK4
HRAS
PRKDC
FANCG
CNOT3
RNF43
COL5A3
CTDP1
XPO1
TSC2
APC
TAF1L
UNC13D
COL15A1
MYBBP1A
BRIP1
KEAP1
NCOR1
ERCC2
ZBTB16
ITSN1
TSC1
MED12
BRPF1
HDAC4
KMT2C
GNAS
ZMYM3
BAG4
STAB2
WASF3
FANCD2
FANCA
ZFP36L2
LSAMP
USP28
IGF2R
KDM6A
CDKN1B
IDH1
USP7
CHD3
FOXA1
FER
MATN4
ADAMTS8
MIRLET7BHG
CDK12
SETDB1
ATR
SAMD9
RSPO2
RAD51D
PYHIN1
BLM
PTPRC
TBC1D2
PRPF8
TCF12
PMS1
ZFHX3
PIK3CB
PALB2
NOX3
TTC28
SF3B1
ERF
U2AF1
ARID4A
CDH1
BRCA1
CUL3
ATM
CDC27
ZNF292
DHX30
EHHADH
TCERG1
SSR1
SPOP
SMARCAD1
SETD2
SEL1L3
RNF31
RAD51B
PTPRD
PIK3R1
PIK3CA
NF1
NDST2
NBN
MTOR
MSH2
MRE11
KMT2D
JADE2
GPR19
ETV5
ETV4
ELK4
MSH6
Treatment
01115374-TA2
01115374-TC2
01115156-TA1
01115156-TC5
01115157-TA3
01115157-TC3
01115565-TA1
01115565-TC1
Mutation categories
SV gene flanking
SV gene
nonsynonymous SNV
CN Amplification
Multiple
frameshift INDEL
stopgain
promoter
CN Homozygous Del
splicing
Treatment
pre-treatment
post-progression
Same patient
B
AR
PTEN
TMPRSS2
LSAMP
MYC
PTPRD
ERG
FOXA1
ELK4
TP53
ETV1
IL6ST
PRKDC
SEL1L3
Altered mCRPC post−treatment samples (%)
Altered mCRPC pre−treatment samples (%)
0
20
40
60
C
Intragenic AR breakpoints
Enhancer
AR
DTB-124-BL
Copy-Number
64
32
16
8
4
2
1
0
66
66.5
67
67.5
chrX (Mb)
RPKM
12.5
10.0
7.5
5.0
2.5
0.0
E1
E1b
E2
CE4
E3
CE1
CE2
CE3
E4
E5
E6
E7
E8
1
25
1
1
1
9
16
1
2
2
2
1

Figure S3

Figure S3. Comparison of genomic alterations in disease states.

(A) Alteration status of paired samples from the same patients before and after treatment. Known prostate driver genes with alteration status in any of the included samples are shown.

(B) Comparison of rearrangement frequency in different disease states of mCRPC. The known prostate cancer driver genes that were located within 1 Mb to any SRB region are included.

(C) Intragenic deletion event leading to loss of ligand binding domain of *AR* in sample DTB-124-BL. *AR* gene boundary (green) and its enhancer (yellow) are shown; concave arcs, intra-chromosomal SV events; convex arcs, inter-chromosomal SV events. Copy number values represent 10 kB bins and have been tumor purity corrected. The expression values of all known *AR* exons, including both canonical and cryptic ones, are shown in the top panel. In the bottom panel, the number of reads covering the junction sites of two exons are indicated by weighted arcs.

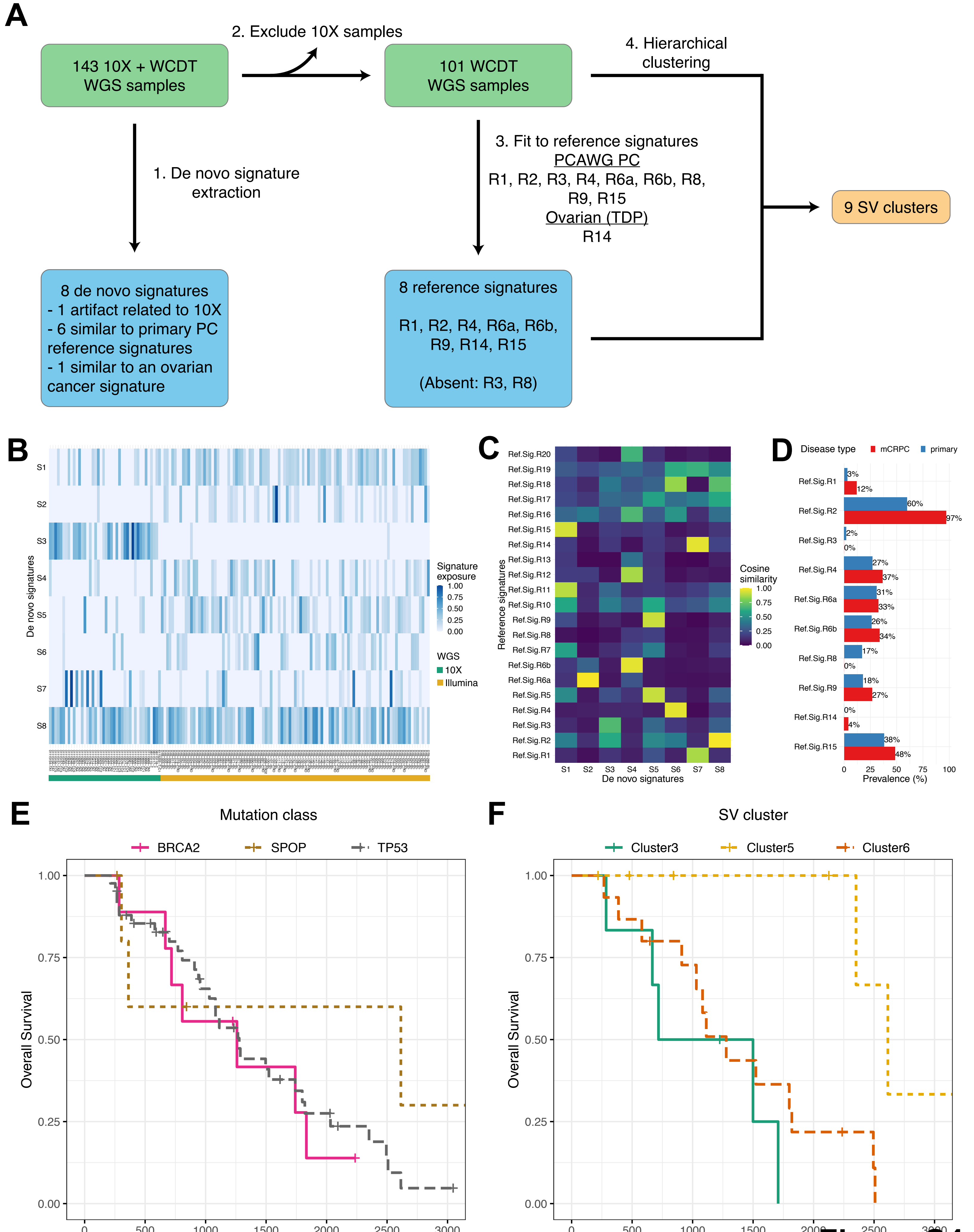
A
143 10X + WCDT WGS samples
2. Exclude 10X samples
101 WCDT WGS samples
4. Hierarchical clustering
1. De novo signature extraction
3. Fit to reference signatures
PCAWG PC
R1, R2, R3, R4, R6a, R6b, R8, R9, R15
Ovarian (TDP)
R14
9 SV clusters
8 de novo signatures
- 1 artifact related to 10X
- 6 similar to primary PC reference signatures
- 1 similar to an ovarian cancer signature
8 reference signatures
R1, R2, R4, R6a, R6b, R9, R14, R15
(Absent: R3, R8)
B
De novo signatures
Signature exposure
WGS
10X
Illumina
C
Reference signatures
De novo signatures
Cosine similarity
D
Disease type
mCRPC
primary
Prevalence (%)
E
Mutation class
BRCA2
SPOP
TP53
Overall Survival
Time (days)
F
SV cluster
Cluster3
Cluster5
Cluster6
Overall Survival
Time (days)

Figure S4

Figure S4. Signature analysis of SV events.

(A) Workflow of SV signature analysis. Samples involved in this analysis are described in green boxes. Details of relevant signatures are shown in blue boxes. The steps for obtaining the final 9 SV clusters are indicated by numbers.

(B) Signature exposure based on de novo SV signatures. The exposure values of each sample were normalized such that the sample-wise sum is 1. Samples are ordered alphabetically based on names. The sequencing technology used for each sample is labeled at the bottom.

(C) Similarity between de novo SV signatures and reference signatures. Pairwise cosine similarity between de novo and reference SV signatures is shown.

(D) Comparison of reference signature (RefSig) prevalence between mCRPC and localized prostate cancer. The prevalence value for a signature in mCRPC was computed based on samples harboring at least 5% signature exposure. Localized prostate cancer prevalence values were obtained from signal.mutationalsignatures.com, which were computed from 199 localized prostate cancer samples from the PCAWG dataset.

(E) Kaplan-Meier curve of prediction using mutation class of key marker genes. Samples were grouped based on the mutation status of the corresponding marker gene.

(F) Kaplan-Meier curve of prediction using SV cluster information. Samples were grouped based on their assignments of the corresponding SV cluster.

SUPPLEMENTAL TABLE LEGENDS

Table S1. Sequencing, clinical and alteration information of all samples involved in this study.

(A) Sequencing metrics of all samples based on linked-read sequencing.

(B) Clinical properties and key genomics metrics of the cohort.

(C) Somatic mutation status of 159 prostate cancer drivers in the cohort. Sample and genes were sorted alphabetically. Genes with no detected mutations were left blank.

(D) Significantly mutated genes ($q \leq 0.1$) detected by dN/dS algorithm.

(E) Somatic copy-number alteration status of 159 prostate cancer drivers in the cohort.

(F) Recurrent copy-number alteration peaks detected by GISTIC.

(G) Gene transecting rearrangements of 159 prostate cancer drivers in the cohort. Types of rearrangement events were included.

(H) Gene flanking rearrangements of prostate cancer drivers in the cohort.

(I) TITAN copy number segments for all samples. Columns with "Corrected_*" were used for analysis in this study.

(J) TITAN optimal solutions selected for all samples.

(K) Structural variant calls for all samples. For samples with linked-read data ("CRPC10X"), union set of detected calls from SvABA, GROC-SVS, and Long Ranger are indicated. `SV.Filter` indicate SV events after filtering. `support` contain evidence from various callers; manual curation of events is indicated here. `CN_overlap_type` contain the final SV classification after annotation with copy number information.

(L) TDP status, copy number gain event counts, and median tandem duplication lengths for all samples.

(M) SV statistics by type in the mCRPC cohort separated by sequencing technology (n=42 for linked-read WGS and n=101 for standard WGS). Numbers shown are median count of SV per sample within sub-cohort of the same sequencing technology.

Table S2. Significantly recurrent breakpoint regions and ETS fusion.

(A) Significantly recurrent breakpoints (SRB) regions ($q \leq 0.1$) in the mCRPC cohort of 143 samples.

(B) Significantly recurrent breakpoints (SRB) regions ($q \leq 0.1$) in the PCAWG localized prostate cancer cohort of 278 samples.

(C) Coefficients and corresponding statistical significance of regression analyses in the mCRPC cohort (n=143), PCAWG localized prostate cancer (n=278) and ICGC early onset prostate cancer (n=253).

(D) Fusion status of the ETS family genes. Gene expression was normalized to z-score for each gene. Genes with no detected fusion events or available expression data were left blank.

(E) Details of SV associated sample frequencies in each cohort and their enrichment statistical results (fisher's exact test) between groups for genes overlapping SRB regions.

Table S3. *AR* alteration patterns in the mCRPC cohort.

Table S4. SV signature in the mCRPC cohort.

(A) Matrix of cosine similarity with rows representing reference signatures and columns representing *de novo* signatures.

(B) Exposure of all 8 *de novo* signatures in the cohort. Values were not normalized.

(C) Exposure of 8 chosen reference signatures in the cohort. Values were not normalized.

REFERENCES

1. Viswanathan SR, et al. Structural Alterations Driving Castration-Resistant Prostate Cancer Revealed by Linked-Read Genome Sequencing. *Cell* 2018;174(2):433-447.e19.

2. Armenia J, et al. The long tail of oncogenic drivers in prostate cancer. *Nature Genetics* 2018;50(5):645–651.

3. van Dessel LF, et al. The genomic landscape of metastatic castration-resistant prostate cancers reveals multiple distinct genotypes with potential clinical impact. *Nat Commun* 2019;10(1):5251.

4. Quigley DA, et al. Genomic Hallmarks and Structural Variation in Metastatic Prostate Cancer. *Cell* 2018;174(3):758-769.e9.

5. Van der Auwera GA, O'Connor B. Genomics in the Cloud2020;https://www.oreilly.com/library/view/genomics-in-the/9781491975183/. cited December 10, 2021

6. Carrot-Zhang J, Majewski J. LoLoPicker: detecting low allelic-fraction variants from low-quality cancer samples. *Oncotarget* 2017;8(23):37032.

7. Saunders CT, et al. Strelka: accurate somatic small-variant calling from sequenced tumor-normal sample pairs. *Bioinformatics* 2012;28(14):1811–1817.

8. Martincorena I, et al. Universal Patterns of Selection in Cancer and Somatic Tissues. *Cell* 2017;171(5):1029-1041.e21.

9. Tate JG, et al. COSMIC: the Catalogue Of Somatic Mutations In Cancer. *Nucleic Acids Res* 2019;47(D1):D941–D947.

10. Ha G, et al. TITAN: inference of copy number architectures in clonal cell populations from tumor whole-genome sequence data. *Genome Res* 2014;24(11):1881–1893.

11. Adalsteinsson VA, et al. Scalable whole-exome sequencing of cell-free DNA reveals high concordance with metastatic tumors. *Nat. Commun.* 2017;8(1):1324.

12. Wala JA, et al. SvABA: genome-wide detection of structural variants and indels by local assembly. *Genome Res* 2018;28(4):581–591.

13. Spies N, et al. Genome-wide reconstruction of complex structural variants using read clouds. *Nature Methods* 2017;

14. Gerhauser C, et al. Molecular Evolution of Early-Onset Prostate Cancer Identifies Molecular Risk Markers and Clinical Trajectories. *Cancer Cell* 2018;34(6):996-1011.e8.

15. Cortés-Ciriano I, et al. Comprehensive analysis of chromothripsis in 2,658 human cancers using whole-genome sequencing. *Nat Genet* 2020;52(3):331–341.

16. Baca SC, et al. Punctuated evolution of prostate cancer genomes. *Cell* 2013;153(3):666–677.

17. Barrett T, et al. NCBI GEO: archive for functional genomics data sets--update. *Nucleic Acids Res* 2013;41(Database issue):D991-995.

18. Gene Expression Omnibus: NCBI gene expression and hybridization array data repository - PubMedhttps://pubmed.ncbi.nlm.nih.gov/11752295/. cited January 6, 2022

19. Leinonen R, et al. The sequence read archive. *Nucleic Acids Res* 2011;39(Database issue):D19-21.

20. Li H, Durbin R. Fast and accurate short read alignment with Burrows-Wheeler transform. *Bioinformatics* 2009;25(14):1754–1760.

21. Zhang Y, et al. Model-based analysis of ChIP-Seq (MACS). *Genome Biol* 2008;9(9):R137.

22. Bert SA, et al. Regional activation of the cancer genome by long-range epigenetic remodeling. *Cancer Cell* 2013;23(1):9–22.

23. Du Q, et al. Replication timing and epigenome remodelling are associated with the nature of chromosomal rearrangements in cancer. *Nat Commun* 2019;10(1):416.

24. Taberlay PC, et al. Three-dimensional disorganization of the cancer genome occurs coincident with long-range genetic and epigenetic alterations. *Genome Res* 2016;26(6):719–731.

25. Pomerantz MM, et al. The androgen receptor cistrome is extensively reprogrammed in human prostate tumorigenesis. *Nat Genet* 2015;47(11):1346–1351.

26. Pomerantz MM, et al. Prostate cancer reactivates developmental epigenomic programs during metastatic progression. *Nat. Genet.* [published online ahead of print: July 20, 2020]; doi:10.1038/s41588-020-0664-8

27. Mallick S, et al. The Simons Genome Diversity Project: 300 genomes from 142 diverse populations. *Nature* 2016;538(7624):201–206.

28. Smit, AFA, Hubley, R & Green, P. *RepeatMasker Open-4.0.*. 2013:

29. Davis CA, et al. The Encyclopedia of DNA elements (ENCODE): data portal update. *Nucleic Acids Res* 2018;46(D1):D794–D801.

30. ENCODE Project Consortium. An integrated encyclopedia of DNA elements in the human genome. *Nature* 2012;489(7414):57–74.

31. Ernst J, Kellis M. ChromHMM: automating chromatin-state discovery and characterization. *Nat Methods* 2012;9(3):215–216.

32. Roadmap Epigenomics Consortium, et al. Integrative analysis of 111 reference human epigenomes. *Nature* 2015;518(7539):317–330.

33. Tweedie S, et al. Genenames.org: the HGNC and VGNC resources in 2021. *Nucleic Acids Res* 2021;49(D1):D939–D946.

34. Imielinski M, Guo G, Meyerson M. Insertions and Deletions Target Lineage-Defining Genes in Human Cancers. *Cell* 2017;168(3):460-472.e14.

35. Frankish A, et al. GENCODE reference annotation for the human and mouse genomes. *Nucleic Acids Res* 2019;47(D1):D766–D773.

36. Howe KL, et al. Ensembl 2021. *Nucleic Acids Res* 2021;49(D1):D884–D891.

37. Cerami E, et al. The cBio Cancer Genomics Portal: An Open Platform for Exploring Multidimensional Cancer Genomics Data: Figure 1.. *Cancer Discovery* 2012;2(5):401–404.

38. Gao J, et al. Integrative analysis of complex cancer genomics and clinical profiles using the cBioPortal. *Sci Signal* 2013;6(269):pl1.

39. Uhrig S, et al. Accurate and efficient detection of gene fusions from RNA sequencing data. *Genome Res* 2021;31(3):448–460.

40. Degasperi A, et al. A practical framework and online tool for mutational signature analyses show inter-tissue variation and driver dependencies. *Nat Cancer* 2020;1(2):249–263.
